# Glycan signatures for the identification of cisplatin‐resistant testicular cancer cell lines: Specific glycoprofiling of human chorionic gonadotropin (hCG)

**DOI:** 10.1002/cam4.4515

**Published:** 2022-01-19

**Authors:** Michal Hires, Eduard Jane, Katarina Kalavska, Michal Chovanec, Michal Mego, Peter Kasak, Tomas Bertok, Jan Tkac

**Affiliations:** ^1^ Institute of Chemistry Slovak Academy of Sciences Bratislava Slovakia; ^2^ Translational Research Unit Faculty of Medicine Comenius University and National Cancer Institute Bratislava Slovakia; ^3^ 2nd Department of Oncology Faculty of Medicine Comenius University and National Cancer Institute Bratislava Slovakia; ^4^ Center for Advanced Materials Qatar University Doha Qatar

**Keywords:** cell lines, germ cell tumour, glycans, glycosylation, human chorionic gonadotropin, lectins, testicular cancer

## Abstract

**Background:**

Testicular cancer (TC) is the most frequent type of cancer among young men aged between 15 and 34 years. TC is treated using cisplatin, but 3%–5% of TC patients fail to respond to cisplatin, with a very bad to fatal prognosis. Accordingly, it is most important to quickly and readily identify those TC patients who are resistant to cisplatin treatment.

**Methods:**

This study seeks to investigate changes in the glycosylation associated with cisplatin resistance to TC cell lines.

**Results:**

A specific glycoprofiling of human chorionic gonadotropin (hCG) was analysed in three TC cell lines and one cell line of female origin. A typical calibration curve for hCG glycoprofiling showed a dynamic range up to 50 ng/ml, with a limit of detection of 0.3 ng/ml and assay reproducibility represented by relative standard deviation of 3.0%. Changes in the glycan signatures on hCG were analysed in cisplatin‐sensitive cell lines and in their cisplatin‐resistant sub‐lines using an enzyme‐linked lectin assay (ELLA) protocol. An immobilised antibody was applied to a selective capture of hCG from a cytoplasmic fraction of cell lysates with final incubation using a lectin from a panel of 17 lectins.

**Conclusion:**

The results suggest that one particular lectin *Dolichos biflorus* agglutinin (DBA) can selectively discriminate sensitive TC cell lines from resistant TC cell lines. Moreover, there are additional lectins which can provide useful information about the strength of cisplatin resistance.

## Graphical abstract

This is the first study focused on the use of a specific glycoprofiling of hCG using a panel of lectins for the investigation of glycosylation changes associated with TC or associated with cisplatin resistance. We identified DBA lectin as the most perspective one to evaluate resistivity of TC lines. Moreover, we found some lectins, which can be applied for positive correlation between lectin binding to hCG and cisplatin IC_50_ values for the sensitive cell lines and for the resistant cell lines.

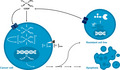

## 1 INTRODUCTION

Testicular cancer (TC) is the most frequent type of cancer among young men aged between 15 and 34 years and represents 1.5% of all cancer types in men and 5% of urological tumours overall.^1^ The current annual TC incidence is 10 new cases *per* 100,000 men in Western countries with an annual increase of up to 6% in Caucasian populations^1^ and with a projected annual incidence of 85,635 new cases worldwide by 2040.^2^ This is due to environmental risk factors, together with a genetic contribution.^3^ More than 90%–95% of TC are germ cell tumours (GCTs) affecting testicular germ cells (cells making sperms).^4^ If the ultrasonography examination provides result, which is consistent with presence of a tumour, the diagnosis is generally confirmed with an inguinal orchiectomy (i.e. removal of one or both testicles and the full spermatic cord) and depending on the TC stage radiotherapy or chemotherapy is offered to the patient.^5^

Cisplatin (*cis*‐diamminedichloroplatinum(II)), as one of the very first metal‐based chemotherapy drugs, is still widely used to treat patients with various types of cancer including TC. This drug is also effective in curing TC patients having metastases with cure rates of up to 90% and with a general survival rate of up to 95%.^6^ The reason why cisplatin works so effectively in treatment of GCTs is that such cells produce the embryonal stem cells. In turn, damaged embryonal stem cells need to be eliminated through apoptosis in order not to pass on mutations to the next generation.^7^ Loss of this embryonic feature might lie behind the development of cisplatin resistance.^7^

There are 3%–5% of TC patients who fail to respond to cisplatin, with a very bad prognosis, dying from the disease within a few months.^3^ Accordingly, it is most important to identify the TC patients resistant to cisplatin treatment in order to avoid long‐term side effects and overtreatment for such young patients, using alternative treatment.^8^

Cisplatin binds to DNA and creates lesions (i.e. protein–DNA complexes and inter/intra‐strand DNA adducts) which cannot be repaired by the DNA repair mechanisms, resulting in a disruption of synthesis of DNA, mRNA and proteins, promoting the accumulation of reactive oxygen species; activating signalling pathways and finally resulting in cell death.^3^ Cisplatin resistance can also be studied on the basis of the order of events followed by introduction of the drug into the body recognising pre‐target mechanisms, on‐target mechanisms and post‐target mechanisms.^3^ Pre‐target mechanisms include those which are active before the drug reaches its target by a decreased intracellular drug accumulation (i.e. a reduced uptake or an increased efflux by copper transporters) or by the presence of compounds such as glutathione or metallothioneins hindering the action of the drug. On‐target mechanisms are very well studied with identification of DNA repair systems or specific polymerases bypassing DNA adducts. Post‐target mechanisms involve the disruption of various signalling pathways or the action of chaperones such as heat shock proteins.^3^ Several factors appear to be responsible for the cisplatin resistance, which are discussed in detail in the review paper, including the involvement of microRNAs, long non‐coding RNAs or exosomes in cisplatin resistance.^9^

Glycomics is an emerging scientific discipline studying the involvement of glycans (complex carbohydrates) in the physiological and pathological processes including cancer development and progression.^10^, ^11^, ^12^ There is growing evidence that the expression of some specific glycans can lie behind resistance to different chemical and physical treatments (such as radiotherapy).^13^ For example, elevated activity of *N*‐acetylglucosaminyltransferase V (GnT‐V), which synthesises β6GlcNAc branched *N*‐glycans,^14^ along with an elevated level of β6GlcNAc branched *N*‐glycans, were determined in a radioresistant nasopharyngeal carcinoma cell line.^13^ Interestingly, suppression of a glycan synthesis caused the radioresistant cell line to revert to the radiosensitive cell line.^13^ Another enzyme involved in drug resistance (to cisplatin) is core fucosyltransferase (FUT8).^15^ One of the core‐fucosylated proteins was copper transporter 1 (CTR1), an important transporter in regulating the uptake of cisplatin, and such a glycan modification of the transporter protein suppressed cisplatin uptake into the cells.^16^ An enhanced level of FUT8 was also linked to radio‐resistance and a poor prognosis in oesophageal squamous cell carcinoma patients with identification of CD147 protein as being core‐fucosylated.^17^ Another study suggests that enhanced levels of antennary fucosylated *N*‐glycans synthesised by upregulated FUT4 fucosyltransferase are also behind multidrug resistance of breast cancer cells.^18^ Elevated expression of two other glycosyltransferases increases multidrug resistance in human leukaemia cell lines^19^ and cisplatin resistance in a serous ovarian cancer cell line.^20^ There are several other studies describing chemoresistance associated with altered glycans in endometrial cancer cell lines,^21^ head and neck squamous cell carcinoma cell line,^22^ ovarian cancer cell lines,^23^, ^24^, ^25^ ovarian cancer patients,^26^ a chronic myeloid leukaemia (CML) patient,^27^ lung cancer cell lines^28^ and acute lymphoblastic leukaemia cell lines.^29^

We have not yet identified any study focusing on an investigation of glycan changes associated with cisplatin resistance of TC cells lines. Hence, the main aim of this study was to investigate changes in the glycan expression of four different cell lines together with their cisplatin‐resistant sub‐lines. We wanted to investigate glycosylation changes in hCG associated with cisplatin resistance and to identify lectins able to provide information about the presence of cisplatin resistance. The main reason for choosing hCG for glycoprofiling is the fact that the level of hCG is routinely used as a TC biomarker to monitor therapy efficiency.^4^ Furthermore, partial glycosylation of hCG is known and determination of one particular glycan form of hCG (i.e. hyperglycosylated form of hCG) is well established. Moreover, to date, the altered glycosylation of hCG was studied only by investigating one particular change in glycan recognised by the antibody B152 (i.e. type 2 *O*‐glycan on Ser132 of the β‐subunit of hCG), which is known as the hyperglycosylated form of hCG (hCG‐H), in relation to various physiological and pathological conditions.^4^ Thus, the assay presented in the current study based on a specific glycan profiling of hCG using a panel of 17 lectins (Table S1) in an ELISA‐like format of analysis (i.e. enzyme‐linked lectin assay [ELLA]) is wholly new.

## 2 MATERIALS AND METHODS

### 2.1 Reagents

#### 2.1.1 Antibodies

The following antibodies were purchased from Abcam and were used in the study: anti‐hCG beta core fragment antibody [INN‐hCG‐106] (ab11382); anti‐hCG beta 1 epitope antibody [INN‐hCG‐2] (ab11388) and anti‐hCG beta 2 epitope antibody [INN‐hCG‐106] (ab11389).

#### 2.1.2 hCGs

Three different hCG proteins purchased from Abcam were used in the study to identify proper hCG standards to be recognised by the antibody, while being glycosylated: native human hCG beta protein (ab126653), native human hCG protein (ab77874) and recombinant human hCG beta 7 protein (ab164958).

#### 2.1.3 Lectins

The following biotinylated forms of lectins for the enzyme‐linked lectin assays (ELLA) were provided from Vector laboratories: *Aleuria aurantia* lectin (AAL; B‐1395), Concanavalin A (Con A, B‐1005), *Dolichos biflorus* agglutinin (DBA, B‐1035), *Narcissus pseudonarcissus* (Daffodil) lectin (DFL = NPL, B‐1375), *Datura stramonium* lectin (DSL, B‐1185), *Galanthus nivalis* lectin (GNL,B‐1245), *Hippeastrum hybrid* (Amaryllis) lectin (HHL = AL, B‐1385), *Lens culinaris* agglutinin (LCA, B‐1045), *Maackia amurensis* agglutinin II (MAA, B‐1265), *Phaseolus vulgaris* erythroagglutinin (PHAE, B‐1125), *Phaseolus vulgaris* leucoagglutinin (PHAL, B‐1115), Peanut agglutinin (PNA, B‐1075), *Pisum sativum* agglutinin (PSA, B‐1055), *Ricinus communis* agglutinin I (RCA = RCA120, B‐1085), *Sambucus nigra* agglutinin (SNA, B‐1305), *Wisteria floribunda* agglutinin (WFA, B‐1355) and Wheat germ agglutinin (WGA, B‐1025).

#### 2.1.4 Other chemicals

All the following chemicals were obtained from Sigma: bovine serum albumin (lyophilised powder, ≥96%; BSA, A2153), albumin from human serum (lyophilised powder, essentially globulin‐free, ≥99%, A8763), hydrogen peroxide solution (≥30%, for trace analysis, 95321), phosphate‐buffered saline (tablet, P4417), phosphate‐buffered saline with Tween (BioUltra, pH 7.4, 08057) and o‐phenylenediamine (peroxidase substrate, ≥98.0%, OPD, powder, P9029).

Streptavidin‐conjugated HRP (ready‐to‐use) was obtained from Abcam (ab64269).

### 2.2 Surface plasmon resonance (SPR) experiments

#### 2.2.1 Lectins

The following lectins were used to determine whether hCG standard is glycosylated and can be used in a standardisation process within an enzyme‐linked lectin assay (ELLA) format. All the following non‐conjugated lectins were obtained from Vector Laboratories: *Aleuria aurantia* lectin (AAL, L‐1390); *Maackia amurensis* agglutinin II (MAA, L‐1260); *Phaseolus vulgaris* erythroagglutinin (PHAE, L‐1120); *Phaseolus vulgaris* leucoagglutinin (PHAL, L‐1110) and *Sambucus nigra* agglutinin (SNA, L‐1300).

#### 2.2.2 SPR reagents and operation

All the reagents used in SPR experiments were purchased from GE Healthcare, including HBS‐P+ (Buffer 10×; BR‐1006‐71), amine coupling kit (BR100050), EDC (0.4 M), NHS (0.1 M) and ethanolamine hydrochloride (1 M; pH 8.5). For regeneration, the following regeneration buffers were tested (GE Healthcare): NaOH (50 mM; BR‐1003‐58) and glycine/HCl (10 mM; pH 2.5; BR‐1003‐56); acetate 5.0 (BR‐1003‐51) was used for coupling. In order to regenerate the SPR chip following binding with lectins, the following elution solutions were used (Vector Laboratories): that is, solutions for eluting mannose/glucose‐binding lectins (ES‐1100‐100); galactose/GalNAc‐binding lectins (ES‐2100‐100); fucose/arabinose‐binding lectins (ES‐3100‐100); GlcNAc/chitin‐binding lectins (ES‐5100‐100) and sialic acid‐binding lectins (ES‐7100‐100).

SPR assays were run on Biacore X100 (GE Healthcare) using a sensor chip CM5 (29‐1496‐04) under a constant flow rate of 30 μl/min at 25°C. Original SW Biacore X100 Control Software was used to operate the instrument.

#### 2.2.3 Identification of a glycosylated hCG standard and proper anti‐hCG antibody

In this examination, hCG was used as a ligand to be immobilised on the SPR chip. The SPR chip was activated using EDC/NHS (ratio 1+1) amine coupling with the chip activated for 420 s. Then, hCG (ab77874, Abcam) was diluted to 16.3 μg/ml (428 nM) in an acetate immobilisation buffer of pH 5.0 and was immobilised on the CM5 sensor chip for 1000 s. Finally, the SPR chip was blocked with ethanolamine (contact time of 420 s). A typical level of bound hCG was 1735 RU. The chip preparation was completed by washing the cell with a running buffer (HBS‐P+) for 10 min with application of five pulses of 20 mM NaOH for 30 s; finally, the chip was re‐equilibrated with a HBS‐P+ running buffer.

A recombinant hCG (ab164957, Abcam) or a native hCG (ab126653, Abcam) was immobilised on the sensor chip CM5 for 600 s into cell 1 and cell 2, respectively, using a protein concentration of 16.3 μg/ml (428 nM).

Interactions between the ligand immobilised on the chip (hCG) and lectins were measured by a multi‐cycle kinetics mechanism (MCK). Association and dissociation phases were set to 120 and 500 s, respectively. Lectins were diluted in a HBS‐P+ buffer to concentrations of 300, 150, 75, 37.5, 18.8 and 0 nM (lectins at a concentration of 18.8 nM were measured twice). Chip regeneration after each cycle was performed using 60 s pulse with the suitable glycoprotein eluting solution (Vector Laboratories) described above followed by 30 s pulse with 20 mM NaOH; finally, the chip was re‐equilibrated with an HBS‐P+ running buffer.

Interactions between the ligand immobilised on the chip (hCG) and antibodies were also measured by MCK. Sample contact time and dissociation time were set to 120 and 750 s, respectively, for interaction with antibodies diluted in a HBS‐P+ buffer to concentrations of 73.3, 36.7, 18.33, 9.16, 4.58 and 0 nM (antibodies at a concentration of 4.58 nM were measured twice). Chip regeneration after each cycle was performed by surface washing using a 30 s pulse with a glycine/HCl regeneration buffer pH 2.5 and using 30 s pulse with 20 mM NaOH; finally, the chip was re‐equilibrated with an HBS‐P+ running buffer.

In order to double check the bioaffinity interaction between hCG and an antibody, an anti‐hCG antibody was also immobilised onto the SPR chip. An anti‐hCG beta 2 epitope antibody (ab11389, Abcam), diluted to 2.86 μg/ml (19.0 nM) in an acetate immobilisation buffer (pH 5.0) was immobilised to a target level of 400 RU onto the sensor chip CM5 *via* EDC/NHS chemistry and then blocked with ethanolamine (all part of amine coupling kit BR100050, GE Healthcare). Chip preparation was completed by washing its surface for 10 min with a running buffer (HBS‐P+) and by five pulses of 20 mM NaOH for 30 s each. Interactions between antibodies immobilised on the chip and hCG were measured by MCK. Sample contact time and dissociation time were set to 120 s and 750 s, respectively. An analyte was diluted in an HBS‐P+ buffer to concentrations of 239, 119.5, 59.8, 29.9, 14.9 and 0 nM (hCG at a concentration of 14.9 nM was measured twice). Chip regeneration after each cycle was performed by washing the surface with a glycine/HCl regeneration buffer pH 2.5 for 30 s and for 30 s with 20 mM NaOH solution; finally, the chip was re‐equilibrated with an HBS‐P+ running buffer.

Interaction in a sandwich configuration was studied in two different forms by an MCK mechanism. In the first, we used a feature of the Biacore X100 Control Software to start each cycle with a capture. hCG (ab77874) diluted in a running buffer to 81.6 nM was repeatedly captured for 120 s over the immobilised antibody on the CM5 chip. In the next step, the lectin binding was investigated using a common MCK mechanism. In this case, sample (lectin) contact time and dissociation time were set to 120 and 500 s, respectively, and chip regeneration was performed with glycine pH 2.5 for 30 s and 20 mM NaOH for 30 s (i.e. both lectins and hCG were released from the surface). In the second approach, the hCG was injected over the antibody‐immobilised surface just once. Then the lectin binding to hCG captured over the anti‐hCG layer was monitored using MCK. Chip regeneration was performed using a suitable glycoprotein‐eluting solution for 30 s (i.e. only the lectin was released from the surface). In both versions, the lectin concentrations investigated were 300, 150, 75, 37.5, 18.8 and 0 nM.

The data thus obtained were evaluated using the original SW Biacore X100 Evaluation Software with pre‐set preferences.

### 2.3 Enzyme‐linked lectin assays (ELLA)

The ELLA assay was based on a common protocol of a conventional sandwich ELISA. Briefly, 100 μl of an anti‐hCG antibody diluted to 1 μg/ml with PBS was added into the wells of the plate (Nunc Module Plate Nunc Immuno MaxiSorp, Thermo Fisher Scientific, F16 467466). After overnight incubation in a refrigerator, the plate was washed three times with 215 μl of PBST buffer. Unoccupied places in wells were blocked with 200 μl of HSA (1 mg/ml) for 45 min using a plate shaker (Mini‐shaker Multi Bio 3D; Biosan; BS‐010125). The ELISA plate was then incubated with 100 μl of sample and, after washing, 100 μl of lectins diluted by 1 mg/ml of HSA solution to concentration of 1.5 μg/ml were added and incubated for 1 h under shaking. After washing, 100 μl of 75× diluted streptavidin HRP in HSA (1 mg/ml) was added to the wells and incubated for 60 min. Then the plate was rinsed 3× with 215 μl of PBST buffer and once again with PBS. The detection was accomplished using OPD (*o*‐phenylenedimamine) as a substrate. OPD and hydrogen peroxide were dissolved in a citrate‐phosphate buffer (pH = 4.6) to concentrations of 7.4 mM and 0.52 M, respectively. To each well, 100 μl of this OPD‐peroxide solution was applied. The absorbance at 450 nm was measured after 15 min incubation in dark and the reaction was stopped with 3.6 M H_2_SO_4_.

A typical calibration curve showed a dynamic range for detection of hCG up to 50 ng/ml, with a limit of detection of 0.3 ng/ml and assay reproducibility represented by relative standard deviation of 3.0% (0.73%–5.4%).

### 2.4 Oxidation of anti‐hCG antibody

Since lectins are able to bind to the glycans present in the Fc fragment of the antibody (i.e. usually bi‐antennary glycans terminated with sialic acid, galactose or GlcNAc), it is advisable to de‐activate such a glycan recognition site by glycan oxidation.^30^

Glycans of anti‐hCG antibodies were modified to prevent binding of lectins, which would interfere with the glycoprofiling of hCG. Prior to oxidation, the following two reaction solutions were prepared: solution A (150 mM sodium acetate of pH 5.5) and solution B (25 mM sodium metaperiodate in solution A). An antibody ab11389 was diluted to 0.1 mg/ml with solution B and incubated at 4°C in dark for 30 min. The solution was then desalted using previously equilibrated columns (Zeba Spin 7000 MWCO). Freshly prepared 2 mM propionic acid hydrazide in solution A was added to an antibody solution at a 1:1 (v/v) ratio. The reaction mixture was incubated at ambient temperature in dark for 2 h. The final desalting was performed using a new desalting column and the oxidised and blocked antibody was stored at −80°C in aliquots. ELLA was used to control efficiency of oxidation of anti‐hCG antibody glycans. The wells of the ELISA plate were incubated with intact and chemically oxidised antibodies. The surface was subsequently blocked and left to interact with a panel of 17 biotinylated lectins. After 60 min incubation with streptavidin peroxidase (HRP) conjugate and OPD/H_2_O_2_ solution, the signal was read at 490 nm after a blank subtraction.

### 2.5 Cell cultures

Four cisplatin‐sensitive TC cell lines (JEG‐3, NCCIT, NTERA‐2 and TCam‐2) and their cisplatin‐resistant variants (JEG‐3 CisR (D) VOL II, NCCIT CisR (D) VOL II, NTERA‐2 CisR and TCam‐2 CisR) together with a control non‐cancerous testis cell line (Hs 1.Tes) were used in the study.

The TCam‐2 human seminoma cell line (kindly provided by Dr. Kitazawa, Ehime University Hospital) as well as the human embryonal carcinoma cell line NCCIT (ATCC^®^ CRL‐2073™) were maintained in RPMI 1640 medium (GIBCO^®^ Invitrogen) containing 10% FBS, 10.000 IU/ml penicillin, 5 μg/ml streptomycin, 2.5 μg/ml amphotericin and 2 mM glutamine. The human embryonal carcinoma cell line NTERA‐2 (ATCC^®^ CRL‐1973™) and the JEG‐3 choriocarcinoma cell line (ATCC^®^ HTB‐36™) were cultivated in high‐glucose (4.5 g/L) DMEM (PAA Laboratories GmbH) supplemented with 10% FBS (GIBCO^®^ Invitrogen), 10.000 IU/ml penicillin (Biotica), 5 μg/ml streptomycin, 2.5 μg/ml amphotericin and 2 mM glutamine (PAA Laboratories GmbH). Finally, the human normal testis cell line Hs 1.Tes (ATCC^®^ CRL‐7002™) was grown in high‐glucose (4.5 g/L) DMEM (PAA Laboratories GmbH) with the addition of 10% FBS (GIBCO^®^ Invitrogen).

Cisplatin‐resistant sub‐lines (JEG‐3 CisR (D) VOL II, NCCIT CisR (D) VOL II, NTERA‐2 CisR and TCam‐2 CisR) were generated in our laboratory over a period of 6 months. Cisplatin‐resistant sub‐lines used in this study were derived by a long‐term propagation of matched parental cells in sub‐lethal concentrations of cisplatin (Hospira UK Ltd., Queensway Royal Leamington Spa) in the culture medium described previously.^31^ TC cells in the exponential growth phase were initially exposed to 0.05 µg/ml cisplatin. When the cells started to expand, the cisplatin concentration was gradually increased to 0.1 µg/ml. Subsequently, de novo derived cisplatin‐resistant sub‐lines were continuously maintained in 0.1 µg/ml cisplatin in culture media.

All the used parental as well as cisplatin‐resistant cell lines were incubated at 37°C in a humidified atmosphere with 5% CO_2_. The characteristics of the cell lines investigated are summarised in Table S2.

### 2.6 Measurement of cell viability and determination of IC_50_ values against cisplatin

For the determination of chemosensitivity, cisplatin‐sensitive and cisplatin‐resistant TC cells of 2 × 10^3^ cells/100 µl (for NTERA‐2, TCam‐2 and JEG‐3 cell line) and of 4 × 10^3^ cells/100 µl for NCCIT cell line, respectively, per well, were placed in 96‐well white‐walled plates (Corning Costar Life Sciences) and treated with cisplatin (0.01–5 µg/ml) overnight. The relative viability of the cells was measured using the CellTiter‐Glo™ Luminescent Cell Viability Assay (Promega Corporation) according to the manufacturer's protocol and evaluated by the LUMIstar GALAXY reader (BMG Lab Technologies) after 6–7 days (NTERA‐2/NTERA‐2 CisR, TCam‐2/TCam‐2 CisR and JEG‐3/JEG‐3 CisR (D) VOL II cells) or after 3 days of treatment (NCCIT and NCCIT CisR (D) VOL II cells). Each concentration was measured in quadruplicates. IC_50_ values were calculated by CalcuSyn 1.1 software (Biosoft).

### 2.7 Extraction of cytosolic protein fraction

A cytosolic protein fraction from TC cell lines was prepared using Fraction PREP TM Cell Fractionation kit (Bio Vision) according to the manufacturer's protocol. Briefly, 4 x 10^6^ cells (NTERA‐2, TCam‐2 and all the cisplatin‐resistant cell lines) or 8 × 10^6^ cells (JEG‐3 and NCCIT parental cell lines) were centrifuged at 700 × g for 5 min. Pellets of cells were washed with 5–10 ml of ice‐cold PBS and centrifuged again at 700 x g for 5 min. Supernatants were removed and the pellets thus obtained were re‐suspended in 400 µl of Cytosol Extraction Buffer Mix (CEB Mix containing DTT and Protease Inhibitor Cocktail). After 20 min incubation of the samples on ice, the analysed samples were centrifuged again at 700 × g for 5 min. The isolated supernatants, representing the cytosolic protein fraction, were stored at −80°C.

## 3 RESULTS

### 3.1 Assay optimisation

Investigation of the lectin binding to hCG was performed using surface plasmon resonance (SPR) involving a panel of five lectins, three hCG standards and using injections of lectins at seven different concentration levels (i.e. 0; 18.8; 18.8; 37.5; 75; 150 and 300 nM). In total, at least 107 SPR sensorgrams were recorded and evaluated. Recombinant hCG (ab164958) was not glycosylated since SPR did not show any binding to a panel of five lectins (data not shown) and this form of hCG was excluded from further assays. Hence, the glycosylation of only two hCG forms was investigated.

**FIGURE 1:**
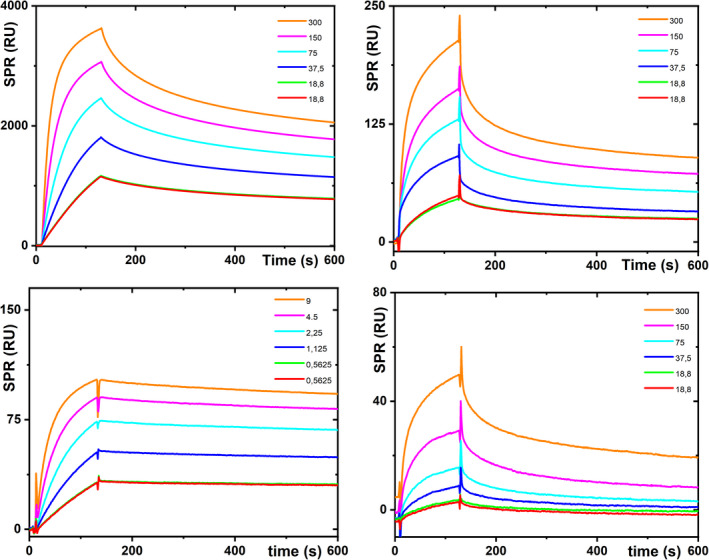
Binding of a PHAE lectin to the immobilised hCG (ab77874; top left figure) and to the immobilised native hCG (ab126653; top right figure). Binding of native human hCG protein (ab77874) to the immobilised anti‐hCG beta 2 epitope antibody (ab11389; bottom left figure) and binding of PHAE lectin to the native human hCG protein (ab77874) captured over the immobilised anti‐hCG beta 2 epitope antibody (ab11389) in a sandwich configuration (bottom right figure)

The results indicate that hCG (ab77874) generally shows a higher level of binding to a panel of lectins (Figure 1 top left), compared to hCG (ab126653) (Figure 1 top right). Two different parameters, extracted from the SPR experiments (i.e. K_D_ and RU_max_) regarding the strength and intensity of the lectin binding to the hCGs immobilised on the SPR chip, are summarised in Table S3 (Supp info file).

In the next experiment, the binding of antibodies to the two glycosylated forms of hCG was investigated. In this particular case, we investigated the binding of three different antibodies at seven different concentration levels (0; 4.58, 4.58; 9.16; 18.33; 36.7 and 73.3 nM) to the two different hCGs immobilised on the SPR chip, that is, investigating at least 42 SPR sensorgrams. Some experiments were performed in a configuration with an antibody immobilised on the SPR chip with hCG present in a running buffer to cross‐check antibody–hCG bioaffinity interactions (i.e. 63 SPR sensorgrams). A summary from these investigations is provided in Table S4. A typical SPR sensorgram for binding of the hCG protein (ab77874) to the immobilised anti‐hCG beta 2 epitope antibody (ab11389) is shown in Figure 1 bottom left.

The results suggest that the best bioaffinity interaction was achieved between the native human hCG protein (ab77874) captured over the immobilised anti‐hCG beta 2 epitope antibody (ab11389) (Table S4).

In the final SPR experiment, we investigated the possibility of binding lectins to hCG (ab77874) bioaffinity captured on the SPR chip modified by the immobilised anti‐hCG beta 2 epitope antibody (ab11389). In other words, it was important to determine whether a biorecognition between hCG and an antibody occurs at the epitope, which is distant from the glycan‐containing epitope on hCG making it possible to form an Ab/hCG/lectin sandwich configuration, which is required for a specific glycoprofiling of hCG.

The results showed that MAA, SNA and PHAE lectins (binding of PHAE lectin is shown in Figure 1 bottom right) were able to bind to hCG bioaffinity captured to the antibody immobilised on the SPR chip. Hence, for the ELLA assays, hCG (ab77874) as a glycoprotein standard and anti‐hCG beta 2 epitope antibody were used in the subsequent assays.

### 3.2 Oxidation of anti‐hCG antibody and its binding performance towards hCG

The binding of lectins to a non‐oxidised (native) and an oxidised anti‐hCG was performed in an ELISA‐like analysis format. The experiment indicates four lectins, in particular, significantly binding to the non‐oxidised anti‐hCG antibody. Of those four lectins, two bind to fucose (AAL and LCA) and the other two lectins bind to mannose glycan units (ConA) or terminal 2,6‐SA (SNA) on the non‐oxidised antibody. Oxidation of the glycan on the anti‐hCG resulted in a significant decrease in binding of all the lectins investigated (Figure S1). On the other hand, oxidation of the glycans on anti‐hCG also resulted in a significant decrease in the bioaffinity of the interaction with hCG from K_D_=0.028 nM for the non‐oxidised antibody form to K_D_=3.7 nM for the oxidised form of the antibody. Hence, oxidation of the antibody significantly reduced the biorecognition affinity by two orders of magnitude, which is in agreement with our previous study.^30^

In the next sequence, we used the oxidised form of the antibody taking into account that the binding of ConA and AAL to the antibody was not fully suppressed. So the results obtained by the glycoprofiling of hCG using ConA and AAL should be treated with caution.

### 3.3 Differences between control cell line and sensitive cancer cell lines

In this study, we used four different cell lines (NCCIT, JEG‐3, TCam‐2 and NTERA‐2) with cisplatin‐sensitive and cisplatin‐resistant variants and one control cell line (CTRL i.e. Hs 1.Tes cell line) (Table S2). We investigated the glycan composition on hCG using a cytoplasmic fraction of cell lysates. A specific hCG glycoprofiling was performed using an optimised assay format with the antibody immobilised on an ELISA plate, followed by incubation with the cytoplasmic fraction of cell lysate and the sandwich configuration was completed by a final incubation with a lectin from a panel of 17 lectins. A summary of the cell lines used in this study accompanied with cisplatin IC_50_ values for sensitive and resistant variants of the cell lines is provided in Table S2.

**FIGURE 2:**
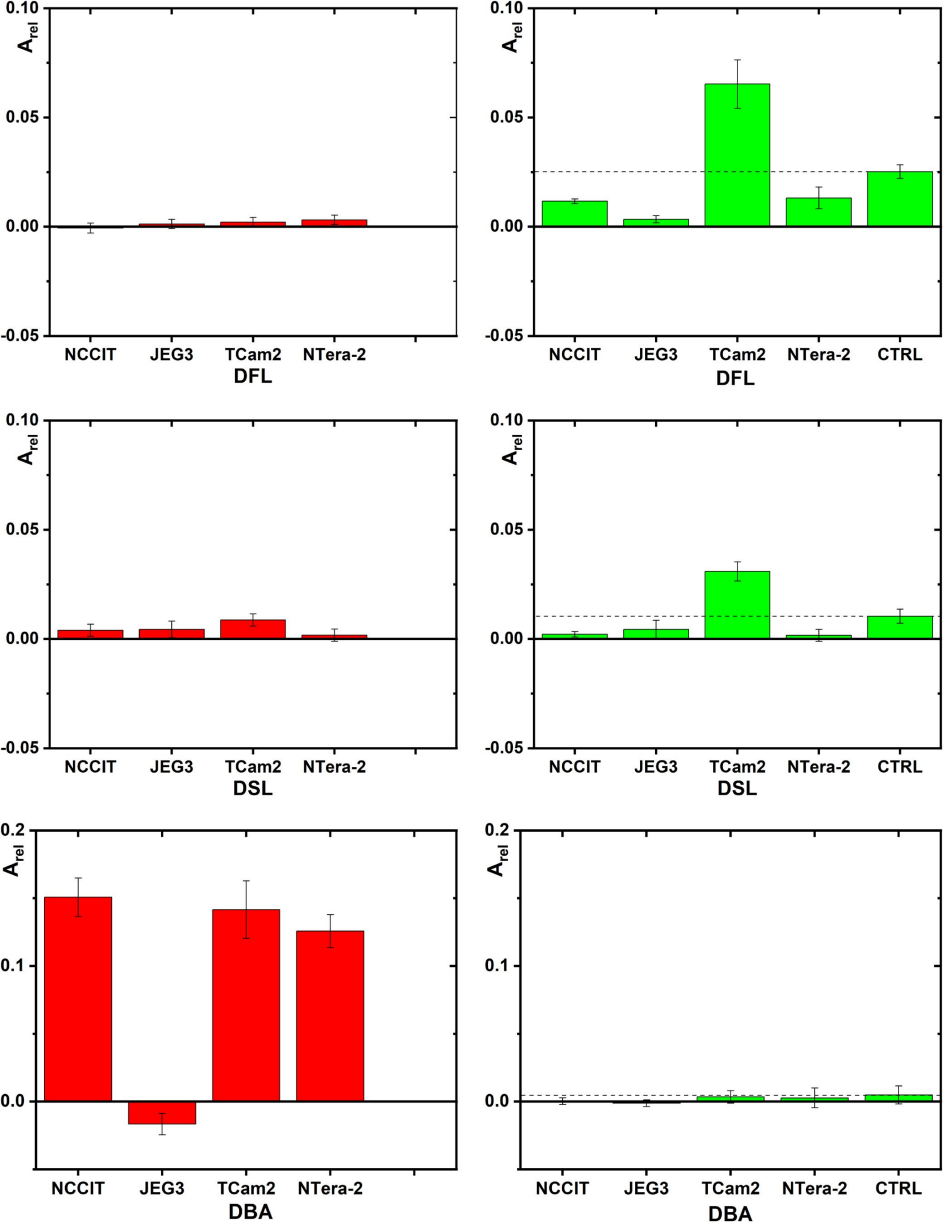
Graphs showing binding of three lectins (DFL, DSL and DBA) to hCG present in the cytosolic fractions of five cell lines (sensitive cell lines shown by green bars) or four cell lines (cisplatin‐resistant cell lines shown by red bars) in an ELLA format of analysis. CTRL = control cell line Hs 1.Tes; DFL = Daffodil lectin; DSL = *Datura* *stramonium* lectin and DBA = *Dolichos biflorus* agglutinin. Binding of additional 14 lectins to hCG present in the cytosolic fraction of sensitive‐ and cisplatin‐resistant cell lines is shown in the Supp. Info file in Figure S1. N.B. the CTRL column is missing for the resistant cell lines simply because we did not possess such a cell line (graphs on the left)

The binding of selected lectins to hCG immunocaptured by the anti‐hCG antibody in the ELLA experiment is shown in Figure 2. A specific glycoprofiling of hCG using the remaining lectins is shown in the Supp. info file (Figure S2). Initially we wanted to investigate glycan changes associated with TC and this is why we compared binding of lectins to glycan present on hCG either in control cell line or in cancerous cell lines.

#### 3.3.1 NTERA‐2 cell line

The NTERA‐2 cell line is a primary male testicular cell line, representing a malignant pluripotent embryonal carcinoma isolated from lung metastases with non‐seminoma‐related cells.^3^

In general, it may be stated that no significant changes in hCG's glycans are observed between the sensitive NTERA‐2 and the CTRL cell lines, when considering mannose‐recognising lectins such as ConA, HHL, DFL, GNL and PSA. Insignificant glycan changes when compared with the CTRL cell line were also observed for other lectins such as DBA, RCA, PNA, WGA, DSL, PHAL and PHAE (Figure 2 and Figure S2).

A significant difference between the sensitive NTERA‐2 and the CTRL cell lines was observed using the LCA lectin (recognising core fucose, cFuc) and the AAL lectin (recognising core and antennary fucose (aFuc)) with such glycans being present in lower amounts in the sensitive NTERA‐2 cell line compared to the CTRL one. Significantly lower binding to hCG from the sensitive NTERA‐2 cell line than for the CTRL was also observed for the WFL, SNA and MAA lectins (Figure 2 and Figure S2).

Accordingly, we may conclude that the sensitive NTERA‐2 cell line does not synthesise a higher level of glycans than the CTRL cell line, which accords with the published data.^32^

#### 3.3.2 TCam‐2 cell line

TCam‐2 cell line represents a testicular seminoma‐like cell line of male origin.^3^

While there is no difference in ConA binding, a significant increase in HHL and DFL binding to hCG for the cancerous‐sensitive cell line over the CTRL cell line was observed (Figure 2 and Figure S2). This indicates the presence of an incomplete glycan composed of short mannose‐containing oligosaccharides, that is, paucimannose (pMan) in a higher amount on hCG from the cancerous‐sensitive cell line TCam‐2.

A significant increase in the binding of lectins to hCG from the cancerous‐sensitive cell line over the CTRL cell line was further observed for WFL, AAL, RCA, DSL, WGA, PHAL and SNA.

Hence, we may conclude that there is an increased expression of glycans containing pMan (HHL and DFL), aFuc (AAL), GalNAc (WFL), Gal/GalNAc (RCA), Gal‐GlcNAc (DSL), GlcNAc (WGA), branched *N*‐glycans (PHAL) or 2,6‐SA (SNA)‐containing glycans in the sensitive TCam‐2 cell line than in the CTRL cell line. An increased binding of SNA to hCG from the cancerous cell line TCam‐2 over the control cell line is in agreement with findings in the literature, where increased levels of sTn and sT antigens were shown in the tissues of the patient with seminoma type of TC.^32^

Analysis of *N*‐glycans in TC serum samples performed by another group of researchers revealed that, in particular, levels of tri‐/tetra‐antennary glycans (PHAL) with terminal sialylation (SNA and/or MAA), galactosylation (RCA and/or DSL) or terminal GlcNAc (WGA), were increased in GCT patients.^33^ This accords with the results obtained for the TCam‐2 cell line with high binding towards hCG observed in our study for the HHL, DFL, AAL, WFL, RCA, DSL, WGA, PHAL and SNA lectins for hCG from the cancerous‐sensitive cell line compared to the CTRL cell line.

#### 3.3.3 JEG‐3 cell line

JEG‐3 cell line represents choriocarcinoma of placenta cell line of female origin.

The JEG‐3 cell line exhibits lower ConA (Man), DFL (pMan), GNL (Man), LCA (cFuc), AAL (aFuc), WFL (GalNAc) and SNA (2,6‐SA) binding than in the CTRL cell line (Figure 2 and Figure S2). The glycan expression profile for the JEG‐3 cell line is similar to the NTERA‐2 cell line, which also expressed a lower level of glycans than the CTRL cell line. The presence of fucosylated and 2,6‐SA‐containing glycans on hCG in the JEG‐3 cells accords with the literature data.^34^

#### 3.3.4 NCCIT cell line

The NCCIT cell line represents mixed non‐seminoma cells with a pluripotent embryonal carcinoma (teratocarcinoma) of male origin.^3^ The protein hCG from the cell line exhibits lower binding than the CTRL cell line for several lectins such as ConA (Man), DFL (pMan), LCA (cFuc), AAL (aFuc) and SNA (2,6‐SA) with a slight increase observed for HHL (pMan) (Figure 2 and Figure S2).

Hence, a lower expression of various types of glycans on hCG from the NCCIT cell line than for the CTRL cell line is similar to the JEG‐3 and NTERA‐2 cell lines.

### 3.4 Differences between sensitive and cisplatin‐resistant cancer cell lines

The binding of selected lectins to hCG immunocaptured by the anti‐hCG antibody in the ELLA experiment is shown in Figure 2. A specific glycoprofiling of hCG by the remaining lectins is shown in the Supp. Info file (Figure S2).

#### 3.4.1 NTERA‐2 cell line

There is a decrease in ConA, HHL and DFL (Man or pMan) binding to hCG from the resistant NTERA‐2 cell line when compared with the sensitive NTERA‐2 cell line with a significant increase in DBA (Sd^a^ antigen, GalNAc‐Gal) and SNA (2,6‐SA) binding to hCG expressed by the resistant cell line over the sensitive one (Figure 2 and Figure S2). Other lectins exhibited an insignificant change in binding pattern to hCG produced by the resistant cell line over the sensitive one (Figure S2).

#### 3.4.2 TCam‐2 cell line

The resistant TCam‐2 cell line in comparison with the sensitive cell line exhibits hCG with a significantly lower affinity towards the following lectins: ConA, HHL, DFL, AAL, WFL, DSL, WGA, PHAL and SNA with a significantly increased binding of DBA (Figure 2 and Figure S2).

#### 3.4.3 JEG‐3 cell line

The resistant JEG‐3 cell line expresses hCG with a decrease in DBA, SNA and MAA and an increase in AAL binding for the resistant sub‐line, in comparison with the sensitive cell line (Figure 2 and Figure S2).

#### 3.4.4 NCCIT cell line

The resistant NCCIT cell line exhibits a decreased binding of HHL, DFL, AAL, SNA and MAA lectins for hCG, in comparison with the sensitive cell line. Only an increased binding of DBA to hCG was observed for the resistant cell line, in comparison with the sensitive cell line (Figure 2 and Figure S2).

### 3.5 Correlation between lectin binding to hCG of resistant cell lines with cisplatin IC_50_

It is not determined whether the glycosylation pattern on hCG can be considered as a pre‐target or a post‐target cisplatin‐resistant mechanism. Hence, we investigated a correlation between lectin binding to hCG for both the sensitive (potentially a pre‐target mechanism) and the resistant (potentially post‐target mechanism) cell lines versus cisplatin IC_50_ values.

#### 3.5.1 The sensitive cell lines

**FIGURE 3:**
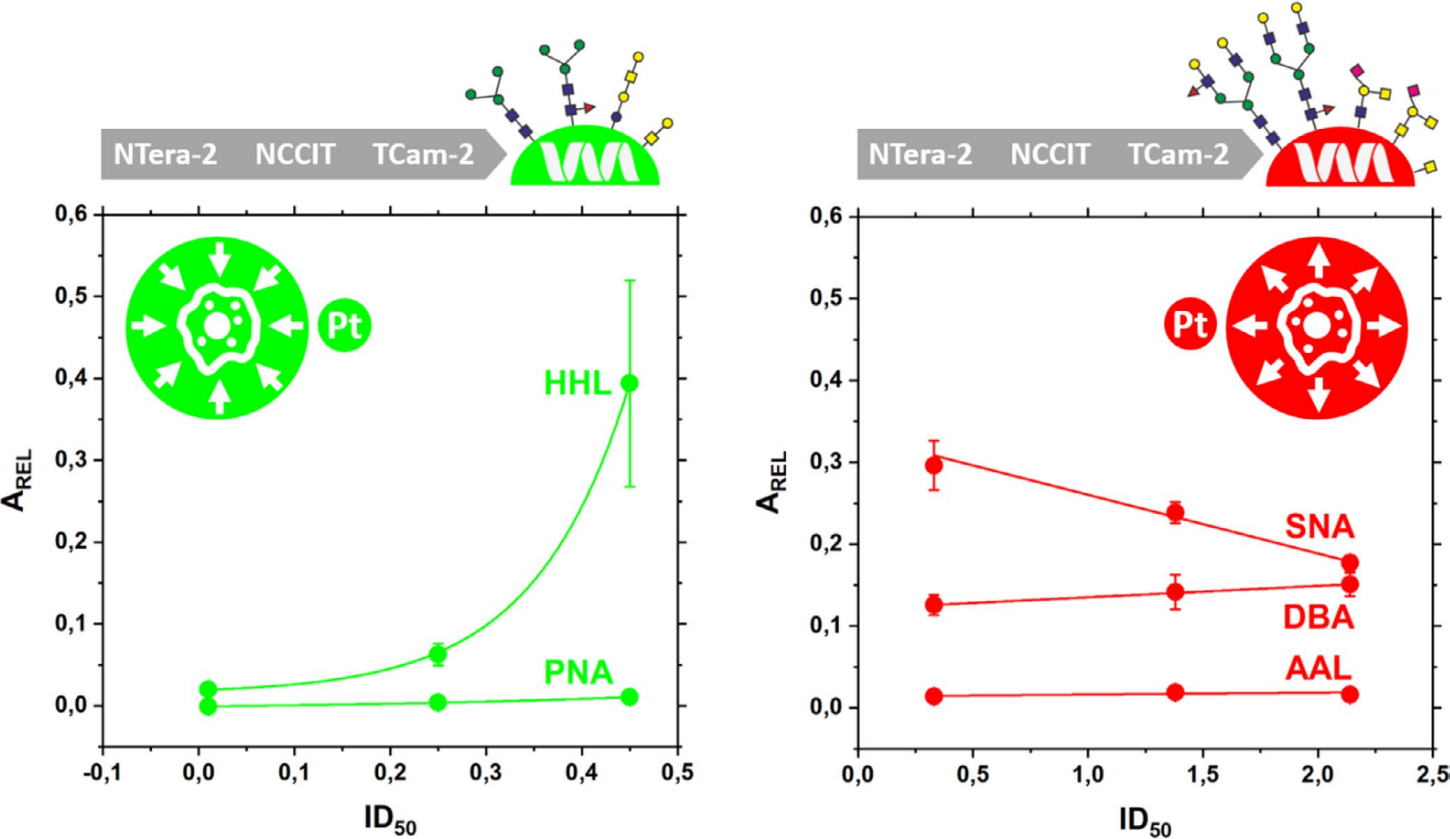
Correlation of the lectin binding to hCG present in the cytoplasmic fraction of sensitive cell lines with the IC_50_ value for two lectins (left) and the lectin binding to hCG present in the cytoplasmic fraction of resistant cell lines with the IC_50_ value for three lectins (right)

A typical correlation between lectin binding to hCG produced by the sensitive cell line and IC_50_ values is shown in Figure 3 left for two lectins (HHL and PNA). There is a positive correlation between the lectin binding to hCG produced by the sensitive cell lines and cisplatin IC_50_ values for the following lectins: AAL, DFL, DSL, HHL, PHAE, PNA, SNA and WFL with a quasi‐exponential and strong pattern of correlation for HHL (Figure 3 left); however, for a lectin such as PNA only a minor change in response with IC_50_ (Figure 3 left) is detected. This might mean that a lectin such as HHL might have a potential for use in predicting a cisplatin resistance in sensitive cell lines (i.e. a pre‐target resistivity mechanism).

#### 3.5.2 The resistant cell lines

If we hypothesise that the changed glycosylation on hCG results from an exposure of cells to cisplatin, then any type of correlation between lectin binding and cisplatin IC_50_ value for the resistant cell lines is of potential value for the identification of TC patients resistant to cisplatin treatment after being exposed to cisplatin for the first time (i.e. after the first round of cisplatin‐based chemotherapy was administered).

DBA lectin binding to hCG showed only a slightly increased pattern with IC_50_ value (Figure 3 right). This is why such a lectin has the potential to be used for examination of the overall cisplatin‐resistant TC cell lines. AAL lectin binding to hCG from the resistant TC cell lines showed only a minor response, that is, only a background signal (Figure 3 right). SNA lectin binding to hCG from the resistant TC cell lines showed a decrease in a lectin binding with IC_50_ value with a potential to apply such a lectin to probe the strength of cisplatin resistance (Figure 3 right). Binding of DBA and SNA lectins to hCG can also be made relative to the binding of AAL lectin to hCG, since the ratiometric signal (i.e. SNA/AAL ratio or DBA/AAL ratio) can provide robust results.

## 4 DISCUSSION

In general, we may conclude that the resistant cell lines express hCG with a significantly higher binding to DBA than the sensitive cell lines. The only exception is the JEG‐3 cell line, which showed a decreased binding of DBA to hCG from the resistant cell line in comparison with the sensitive cell line. This is quite an interesting result since the JEG‐3 cell line is the sole cell line used in this study which is not of a germ cell and not of male origin.

These observations accord well with those in other studies describing downregulated *N*‐acetylgalactosaminyltransferase (B4GALNT2) activity (formation of Sd^a^ antigen) in colon, gastric cancer and embryonal carcinoma.^35^, ^36^, ^37^, ^38^ A reduced activity of B4GALNT2 results in the availability of the glycan precursor (NeuAcα3Galβ4GlcNAc) of Sd^a^ antigen to the enzymatic action of fucosyltransferases expressing sLe^a^ or sLe^x^ antigens on glycans, which are very important glycan determinants of induction of a metastatic process.^35^, ^39^ Thus, an increase in the Sd^a^ level is directly proportional to a decrease in the level of sLe^a^ or sLe^x^ antigens (aFuc) and vice versa.^35^, ^40^ A similar pattern was observed in our study. We may conclude that, in the sensitive cell lines, a low binding of DBA (recognising Sd^a^ antigen) to hCG was observed (Figure 2), while at the same time a high binding of AAL (recognising aFuc‐containing antigens such as sLe^a^ or sLe^x^) to hCG of the sensitive cell line was observed (Figure S2). The trend was reversed for the resistant cell lines with an increased DBA binding (Figure 2) and a decreased AAL binding (Figure S2) for the resistant TC cell lines in comparison with the sensitive cell lines with few exceptions (Figure 2 and Figure S2).

A literature survey revealed that the DBA lectin was able to specifically select and sort glioma‐derived stem cell populations from unsorted tumour cells.^41^ Such a subpopulation had proliferative properties in vitro and a tumour‐forming capability in vivo.^41^ Thus, DBA could be used for the separation of cancer stem cells with a high expression of stemness markers, that is, for isolation of undifferentiated cancer stem cells with a high tumorigenicity and proliferation rate.^41^ The leukaemia cells with a strong binding to DBA overexpress proliferation‐regulated genes and have a low incidence of fragmented nuclei.^42^ An increased expression of B4GALNT2 was also associated with tumourigenesis of lung cancer cells.^43^ Hence, a high binding of DBA to hCG of the resistant TC cell lines might indicate that the resistant TC cell lines might have some of the properties listed above.

In general, it can be concluded that the DBA binding to hCG produced by the resistant cell lines NCCIT, TCam‐2 and NTERA‐2 was significantly higher than in their sensitive counterparts. At the same time, the binding of several lectins to hCG´s glycans was reduced for these four resistant cell lines in comparison with the sensitive cell lines including ConA (NTERA‐2 and TCam‐2), HHL (NTERA‐2, TCam‐2 and NCCIT), DFL (NTERA‐2, TCam‐2 and NCCIT), AAL (TCam‐2 and NCCIT), WFL (TCam‐2), WGA (TCam‐2), SNA (TCam‐2 and NCCIT) and MAA (NCCIT). This is in agreement with the study showing an increased binding of DBA lectin to hepatocellular carcinoma cells treated with the multikinase inhibitor sorafenib, while the binding of lectins such as MAA, PHAL, RCA and SNA was significantly reduced for the cells treated with sorafenib on a lectin microarray using a cytosolic cell fraction.^44^ A cisplatin‐resistant human ovarian cancer cell line exhibited higher binding than a sensitive cell line to three lectins including DBA lectin, when using a lectin microarray using a cytoplasmic cell fraction.^21^

On the other hand, the binding of several lectins to HCG's glycans was lower for the cisplatin‐resistant cell lines than for the sensitive cell lines including SNA lectin. This is in agreement with the study, where it was observed that a resistant serous ovarian cancer cell line exhibited a lower binding of SNA, WFL and seven other lectins.^20^ A decrease in the level of 2,6‐SA was observed in the serum of drug‐resistant patients, as determined by mass spectrometry.^26^ A lectin dot blot using a cytosolic fraction of a head and neck squamous cell carcinoma cell line showed a lower binding of PHAL to proteins with M_w_ = 90–150 kDa for a resistant sub‐line than for a sensitive one.^22^ A similar behaviour was observed in our study, indicating that the amount of tri‐/tetra‐antennary glycans on hCG was lower in the resistant cells than in the sensitive cells.

The level of fucosylated glycans was lower in the resistant ovarian cancer cell line than in the sensitive one, applying mass spectrometry to a cytosolic fraction of cells with released *N*‐glycans.^23^ In general, the same behaviour was observed in this study, with a decreased binding of LCA and AAL lectins to hCG produced by the resistant cells in comparison with the sensitive cells (Figure S2).

It should be observed that all the published studies discussed so far analysed the whole *N*‐ or *O*‐glycome using a lectin microarray or a MS‐based approach. Accordingly, it is important to note that in these studies the authors did not identify proteins with particularly changed glycans. Only one study indicates that a particular glycan signature can be associated with one protein or subset of proteins and the other particular glycan change can be associated with another protein or another subset of proteins.^20^ Zhao et al.^20^ investigated which glycans were overexpressed or underexpressed in resistant serous ovarian cancer cells compared to a sensitive sub‐line using several lectins. A significantly impaired binding of lectins to the glycoproteins of resistant cells, in comparison with sensitive cells, was observed for several lectins such as LCA (increase), ConA (increase), LEL (*Lycopersicon esculentum* lectin, increase in binding) and SNA (decrease), when using a lectin microarray. A lectin dot blot revealed that LCA bound to glycoproteins with M_w_ of 70–180 kDa; ConA bound glycoproteins with M_w_ of 50–180 kDa; LEL bound to glycoproteins with M_w_ of 50–110 kDa, M_w_ of 40–50 kDa, and with M_w_ above 180 kDa; while SNA bound to glycoproteins with M_w_ of 50–180 kDa.^20^ This means that, when comparing a resistant cell line with a sensitive cell line it is most likely that lectins bind glycans that are overexpressed or underexpressed in different glycoproteins. Thus, we may conclude that the most likely glycan changes in hCG associated with resistant cells might differ from the glycan changes in other glycoproteins within resistant TC cells.

**FIGURE 4:**
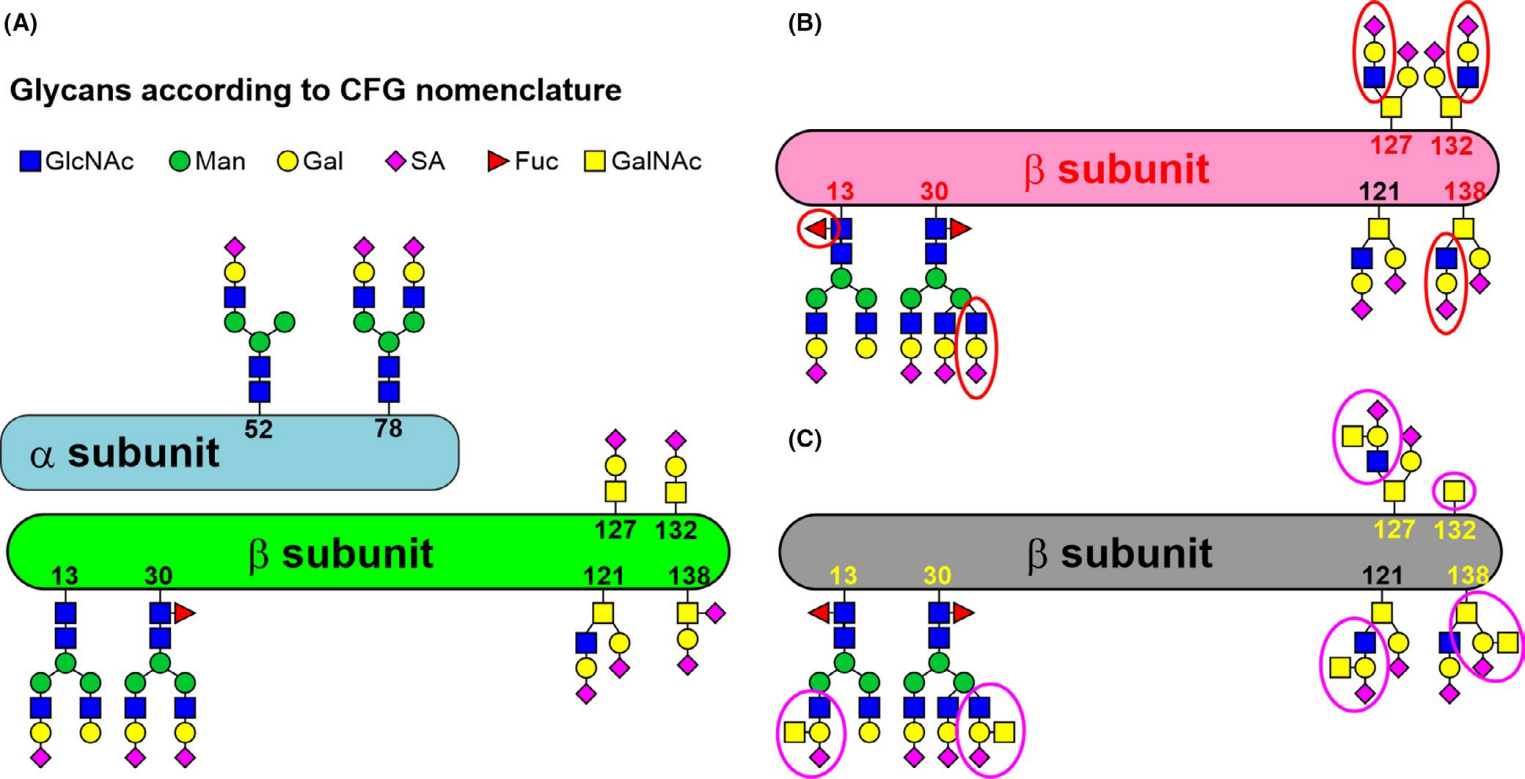
Proposed structure of hCG from healthy cell with hCG composed of two subunits (A), β‐subunit of hCG produced from TC cells (B) and β‐subunit of hCG produced by TC cell line resistant to cisplatin (C). The figure is drawn based on the literature data^4^, ^45^ and on data in this study. In Figure (C) it is possible to see the Sd^a^ antigen at Asn13, Asn30, Ser121 and Ser127, while the CAD antigen is present at Ser138

The results thus obtained lead us to hypothesise that the addition of GalNAc to *N*‐glycans or *O*‐glycans significantly affects the binding of lectins recognising the terminal parts of the glycans (i.e. sialic acid, galactose or branching points) (Figure 4C). Based on data in the literature and the results from this study, we propose the structure of hCG (β‐subunit of hCG) from healthy cells (Figure 4A), cells affected by TC (Figure 4B) or TC cells,^4^, ^45^ which are resistant to cisplatin (Figure 4C) with an expression of several glycan structures recognised by DBA lectin such as Tn antigen, Sd^a^ antigen (i.e. on Ser121) or CAD antigen^46^ (i.e. on Ser138) on hCG produced by the resistant TC cell lines (Figure 4C).

Furthermore, lectins such as AAL, DFL, DSL, HHL, PHAE, PNA, WFL and PSA could potentially be used for the identification of a pre‐target resistant mechanism, while lectin SNA could be used for the identification of a post‐target resistant mechanism (i.e. to identify TC patients resistant to cisplatin treatment after a first round of chemotherapy). This hypothesis, however, needs to be verified using serum/plasma samples of TC patients.

## 5 CONCLUSIONS

This is the first study focused on use of a specific glycoprofiling of hCG using a panel of lectins for investigation of glycosylation changes associated with TC or associated with cisplatin resistance. We identified the DBA lectin as the best prospect for evaluating the resistance of TC lines. Moreover, we identified some lectins to be used for positive correlation between lectin binding to hCG and cisplatin IC_50_ values for the sensitive cell lines and for the resistant cell lines. The results observed in the current study require validation using serum/plasma samples of TC patients in order to corroborate the clinical potential of specific glycoprofiling of hCG to identify cisplatin‐resistant TC patients, who have a poor prognosis.

## CONFLICT OF INTEREST

The authors declare no conflict of interest.

## AUTHOR CONTRIBUTION

**MH** & **EJ**: investigation, data curation and writing (original draft & review and editing); **KK**: investigation, resources and writing original draft; **MC** & **MM**: conceptualisation, resources and writing ‐ review and editing; **PK**: funding acquisition, project administration and writing ‐ review and editing; **TB**: formal analysis, visualisation and writing (original draft & review and editing) and **JT**: conceptualisation, formal analysis, funding acquisition, methodology, supervision and writing (original draft & review and editing).

## ETHICS STATEMENT

The work with TC cell lines did not require ethics approval.

## Supporting information

Supplementary Material

## Data Availability

The data that supports the findings of this study are available in the supplementary material of this article.
