# Supplementary material for: Glycan signatures for the identification of cisplatin‐resistant testicular cancer cell lines: Specific glycoprofiling of human chorionic gonadotropin (hCG)
Source: Cancer Med. 2022 Jan 19;11(4):968–82. doi: 10.1002/cam4.4515 (PMC8855906; doi:10.1002/cam4.4515)
Supplement: Supplementary file 1 — Supplementary Material [file CAM4-11-968-s001.docx]

**Supplementary information**

**Glycan signatures for identification of cisplatin resistant testicular cancer cell lines: specific glycoprofiling of human chorionic gonadotropin (hCG)**

**Michal Hires^1,#^, Eduard Jane^1,#^, Katarina Kalavska^2^, Michal Chovanec^3^, Michal Mego^2,3^, Peter Kasak^4^, Tomas Bertok^1^, Jan Tkac^1,*^**

^1^ Institute of Chemistry, Slovak Academy of Sciences, Dubravska cesta 9, 845 38 Bratislava, Slovakia;

^2^ Translational Research Unit, Faculty of Medicine, Comenius University and National Cancer Institute,

Klenova 1, 833 10 Bratislava, Slovakia;

^3^ 2^nd^ Department of Oncology, Faculty of Medicine, Comenius University and National Cancer Institute,

Klenova 1, 833 10 Bratislava, Slovakia.

^4^ Center for Advanced Materials, Qatar University, P.O. Box 2713, Doha, Qatar

^#^ The authors contributed equally.

^*^ To whom correspondence should be addressed: [Jan.Tkac@savba.sk](mailto:Jan.Tkac@savba.sk); Tel.: 00421 2 5941 0263.

**Table S1:** Specificity for the lectins applied in this study.

| **Lectins** | **Source** | **Glycan specificity** | **Glycan specificity** |
| --- | --- | --- | --- |
| AAL | *Aleuria aurantia* mushrooms | Fucα6GlcNAc (core Fuc), Fucα3(Galβ4)GlcNAc (Le^x^), α3-linked fucose, α4-linked fucose | 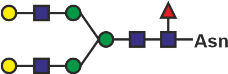 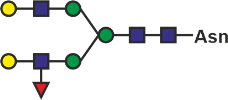 |
| LCA | *Lens culinaris*  agglutinin | α6-linked fucose (core Fuc) of *N*-glycans | *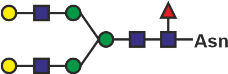* |
| PHAE | *Phaseolus vulgaris* seeds  (erythroagglutinin) | *N*-glycans with outer Gal and bisecting GlcNAc | *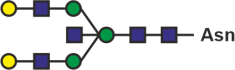* |
| PHAL | *Phaseolus vulgaris* seeds  (leucoagglutinin) | tri/tetra-antennary *N*-glycans | 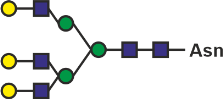 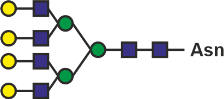 |
| ConA | *Canavalia ensiformis* bean seeds | αMan, αGlc; high-Man; Manα6(Manα3)Man; Manα6Man; Manα3Man | 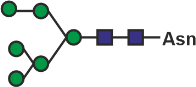 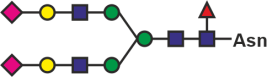 |
| HHL | Hippeatrum hybrid lectin | High-Man type (*N*-linked) | 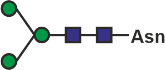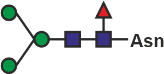 |
| DFL | Daffodil lectin | High-Man including Manα1-6Man | 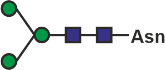 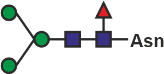 |
| GNL | *Galanthus nivalis* lectin | High mannose type (*N*-linked) | 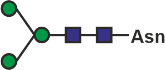 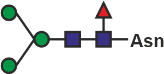 |
| PSA | *Pisum sativum* lectin | High-Man-type *N*-glycans and biantennary *N*-glycans containing core α6Fuc | 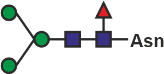 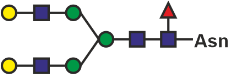 |
| DBA | *Dolichos biflorus* seeds | αGalNAc; terminal GalNAc; GalNAcα3GalNAc, Sd^a^ antigen (*N*-glycans) or CAD antigen (*O*-glycans), Tn antigen | 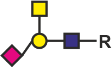 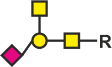 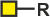 |
| WFL | *Wisteria floribunda* lectin | GalNAc, LacdiNAc, Tn antigen | 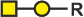 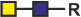 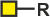 |
| RCA I | *Ricinus communis* seeds | terminal Gal; Galβ4GlcNAc | 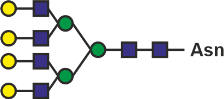 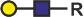 |
| DSL | *Datura stramonium* lectin | *N*-linked (Galβ1-4GlcNAc-)_n_ | 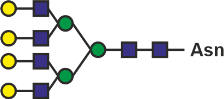 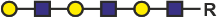 |
| PNA | Peanut agglutinin | Galβ3GalNAc of *N*-glycans, T antigen | 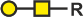 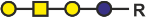 |
| WGA | *Triticum vulgaris* agglutinin | (GlcNAcβ4)_n_, Neu5Ac; poly(*N*-acetyllactosamine) | 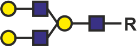 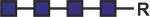 |
| MAA | *Maackia amurensis* seeds agglutinin | Neu5Acα3Galβ4GalNAc; 3-*O*-Suα3Galβ4GalNAc; sT antigen | 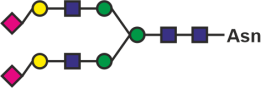 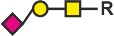 |
| SNA I | *Sambucus nigra* bark agglutinin | Neu5Acα6Galβ4GalNAc; 6-*O*-Suα3Galβ4GalNAc; sTn antigen | 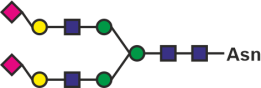 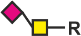 |

Table adapted from our previous study ^1^ with data taken from Vector Laboratories and from Refs. ^2-9^.

**Table S2:** Cell line characteristics ^10^ with IC_50_ values for cisplatin

| **Cell line** | **Disease** | **Origin** | **Sensitive, IC_50_ of CP [μg/mL]** | **Resistant, IC_50_ of CP [μg/mL]** |
| --- | --- | --- | --- | --- |
| **Hs 1.Tes** | normal testis cells, CTRL | M | - | - |
| **JEG-3** | choriocarcinoma | F | 0.29 | 0.95 |
| **NCCIT** | embryonal carcinoma | M | 0.25 | 2.14 |
| **NTERA-2** | embryonal carcinoma | M | 0.01 | 0.33 |
| **TCam-2** | seminoma | M | 0.45 | 1.38 |

**Abbreviations:** **F**: female; **M**: male; **CP**: cisplatin.

**Table S3**: Basic characteristics for binding of lectins to immobilized hCG obtained from the SPR experiments.

| **Lectin** | **hCG (ab126653)** | | **hCG (ab77874)** | |
| --- | --- | --- | --- | --- |
|  | K_D_ [nM] | R_max_ [RU] | K_D_ [nM] | R_max_ [RU] |
| MAA | 74 | 41 | 3.8 | 1770 |
| SNA | 27 | 386 | 74 | 73 |
| AAL | 6.1 | 64 | 140 | 170 |
| PHAE | 11 | 143 | 5.0 | 2940 |
| PHAL | 473 | 29 | 81 | 120 |

**Table S4**: Basic characteristics for binding of antibodies to immobilized hCG as obtained from SPR experiments.

| **Antibody** | **Native hCG (ab126653)** | | **Native hCG (ab77874)** | |
| --- | --- | --- | --- | --- |
|  | K_D_ [nM] | R_max_ [RU] | K_D_ [nM] | R_max_ [RU] |
| ab11382 | 8.4 | 10 | 32 | 5 |
| ab11388 | 1.0 | 29 | 0.75 | 155 |
| ab11389 | 0.045 | 38 | 0.028 | 651 |

**
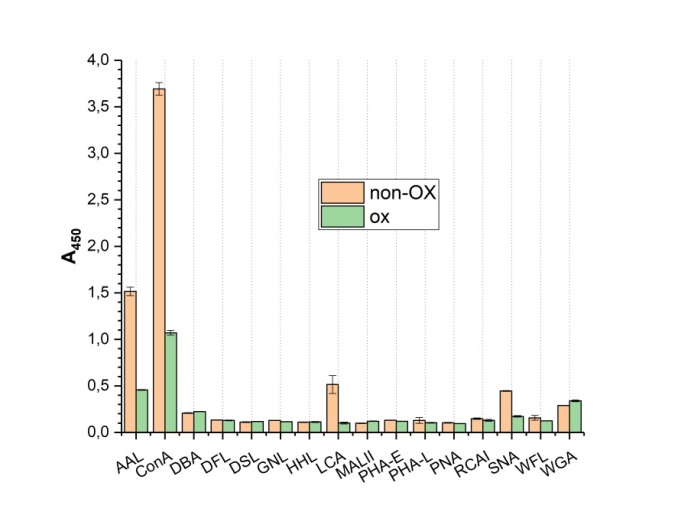
**

**Figure S1**: Binding of lectins to oxidized (ox) and native (non-OX) anti-hCG beta 2 epitope antibody examined in the ELISA-like format of analysis.


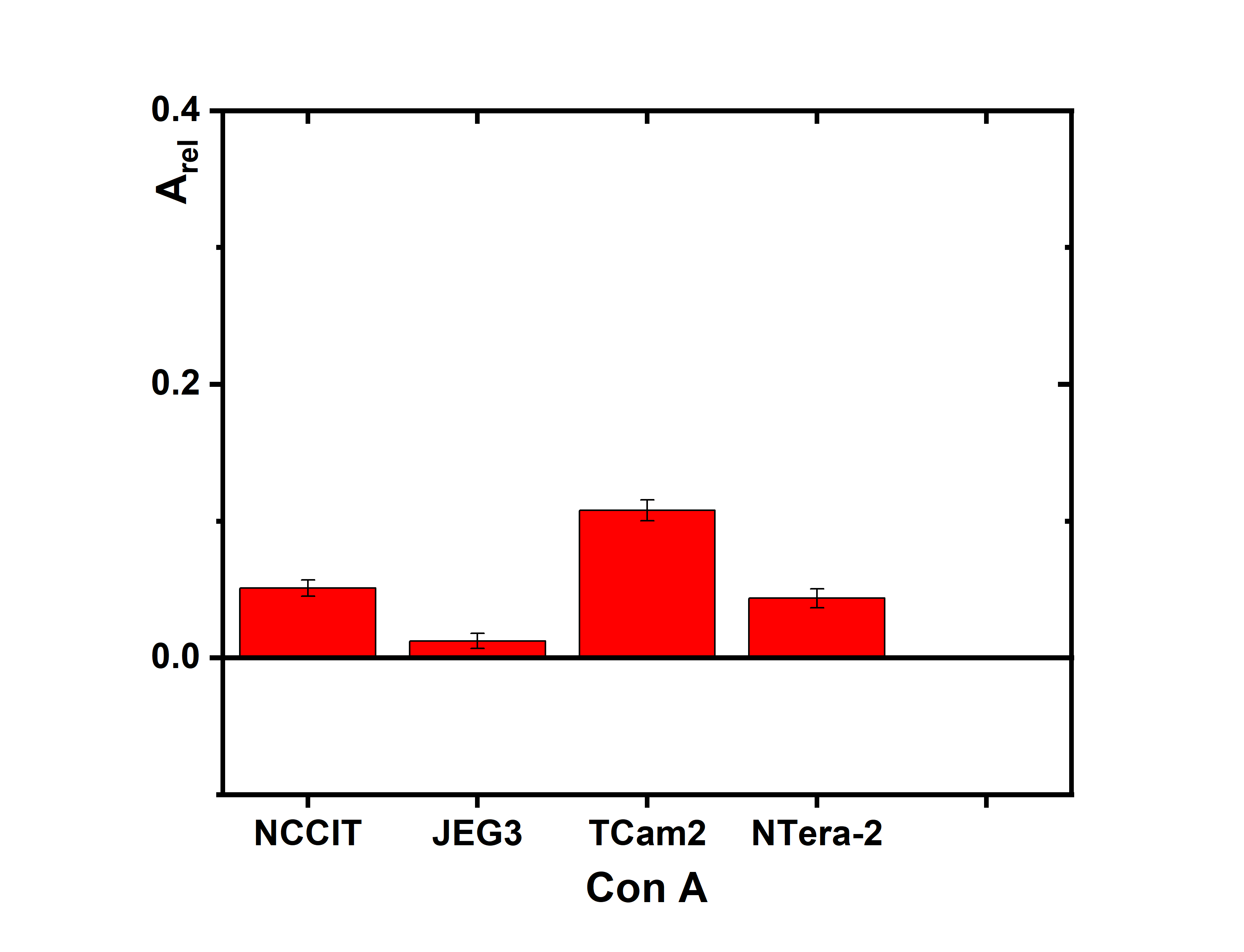

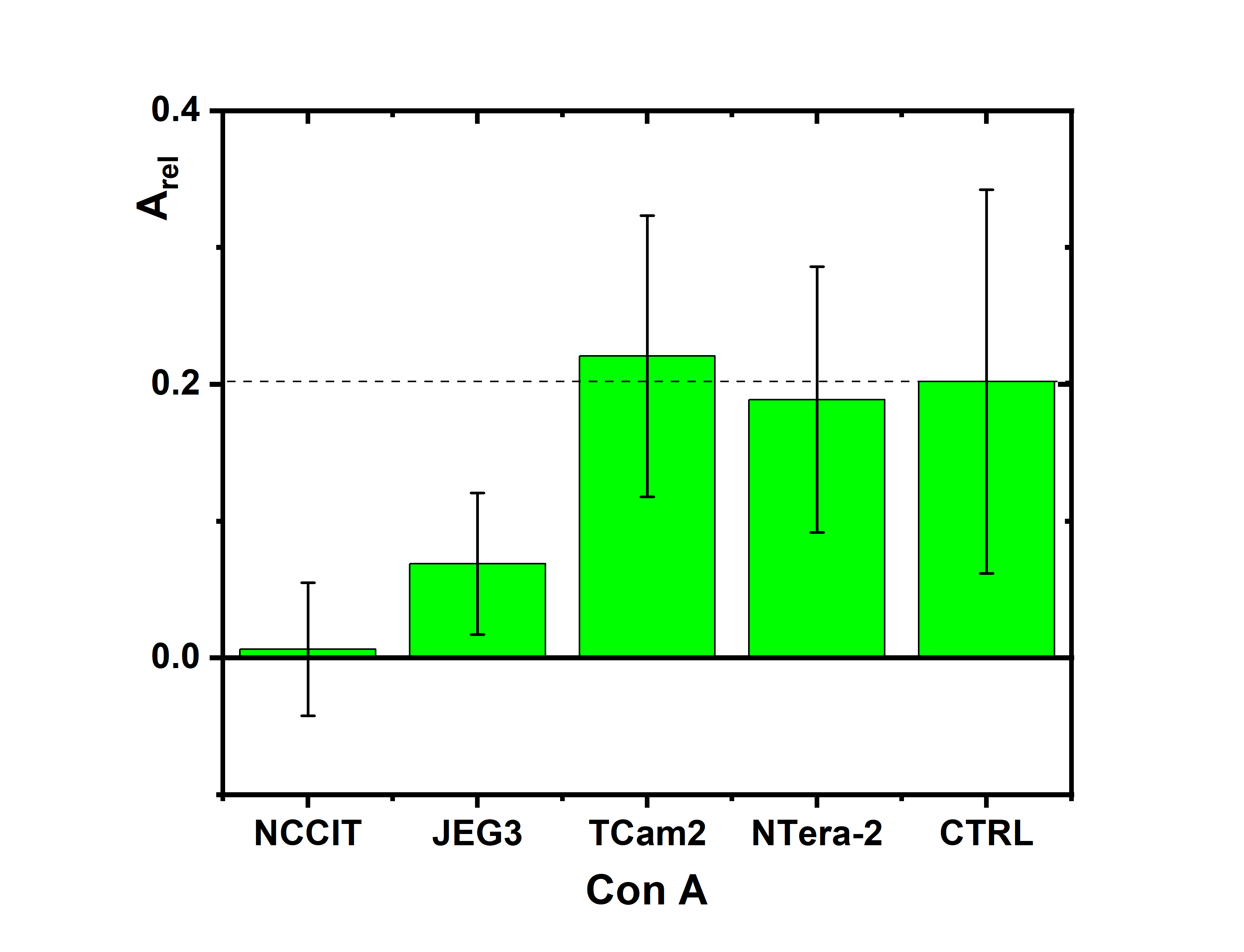


**
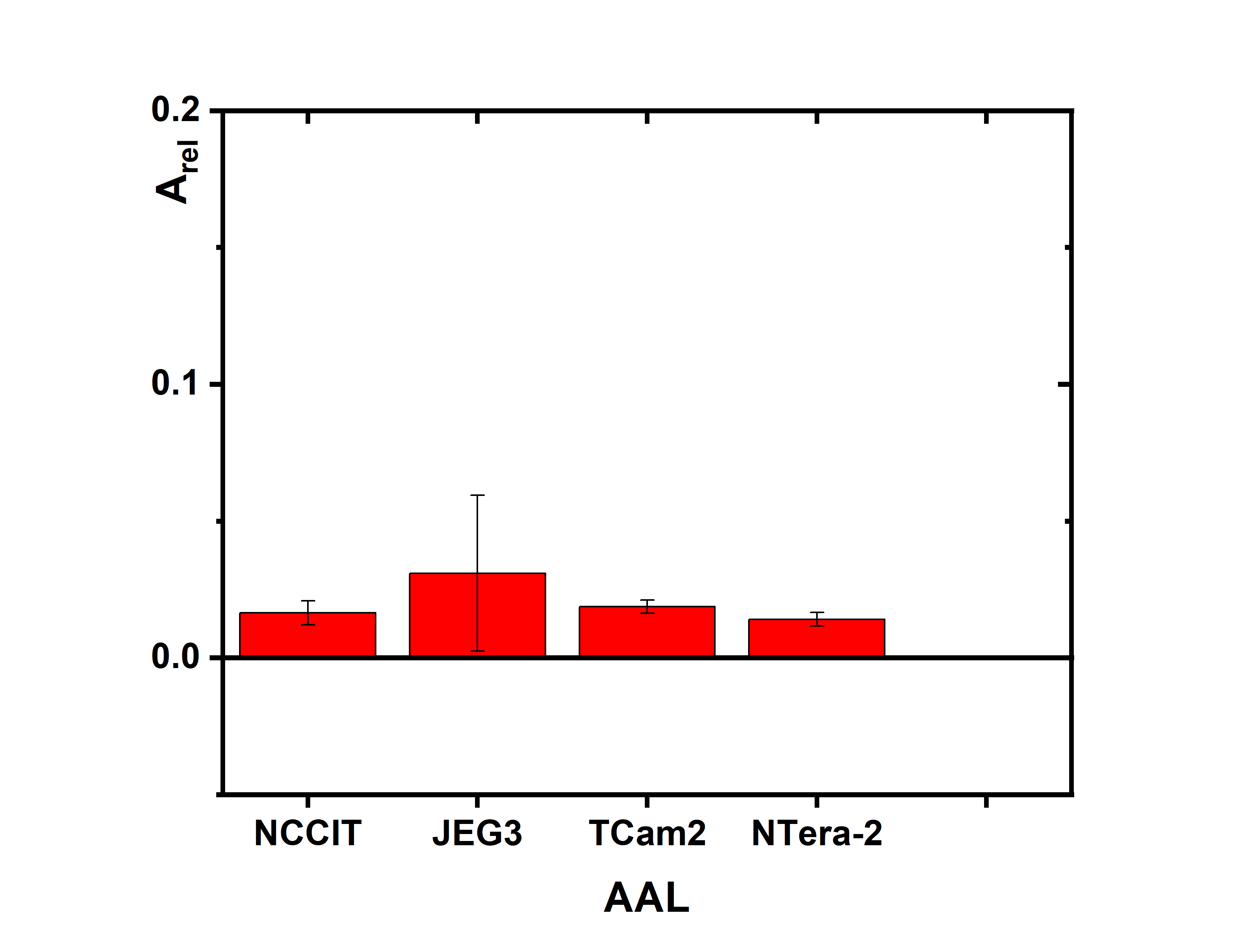

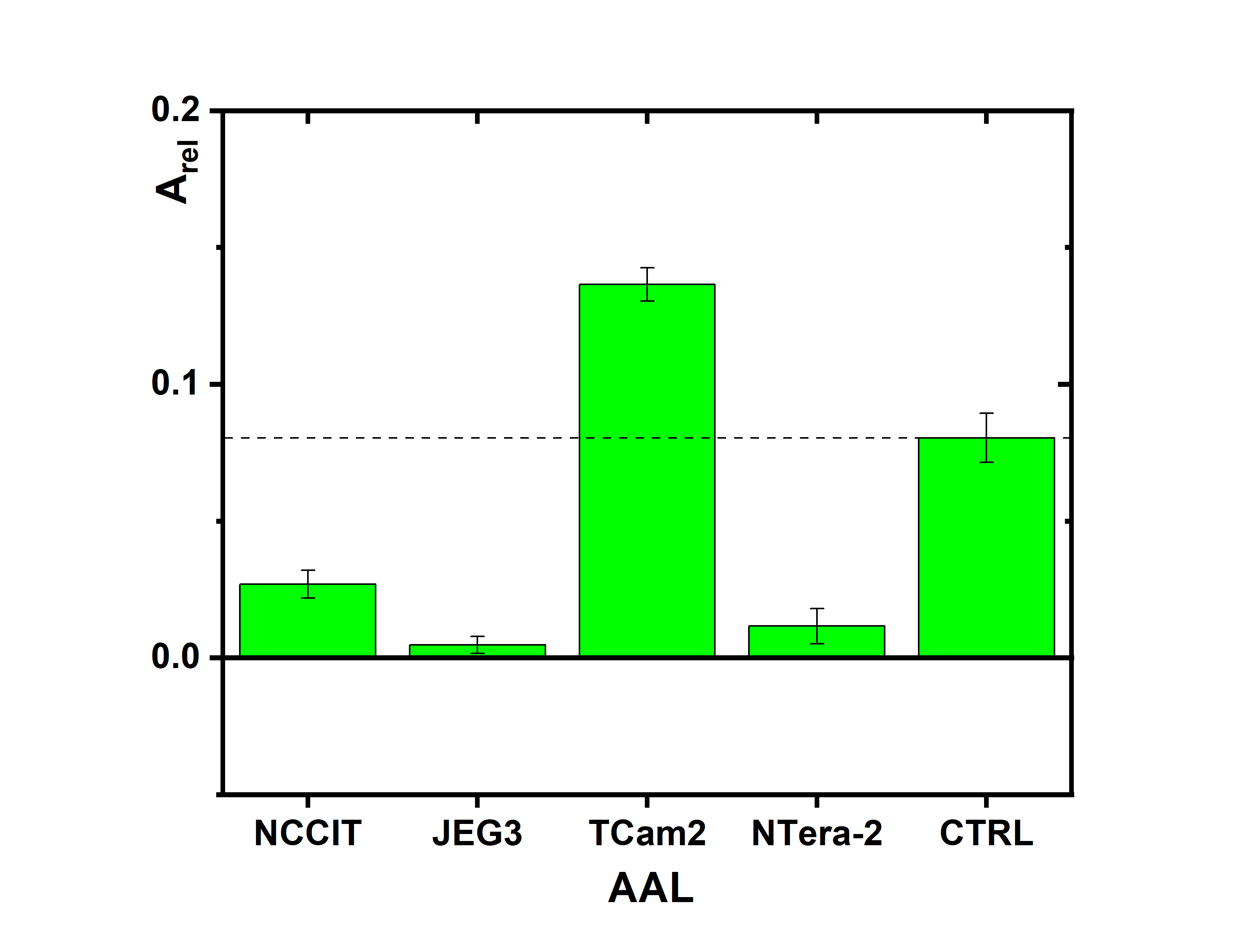
**

**
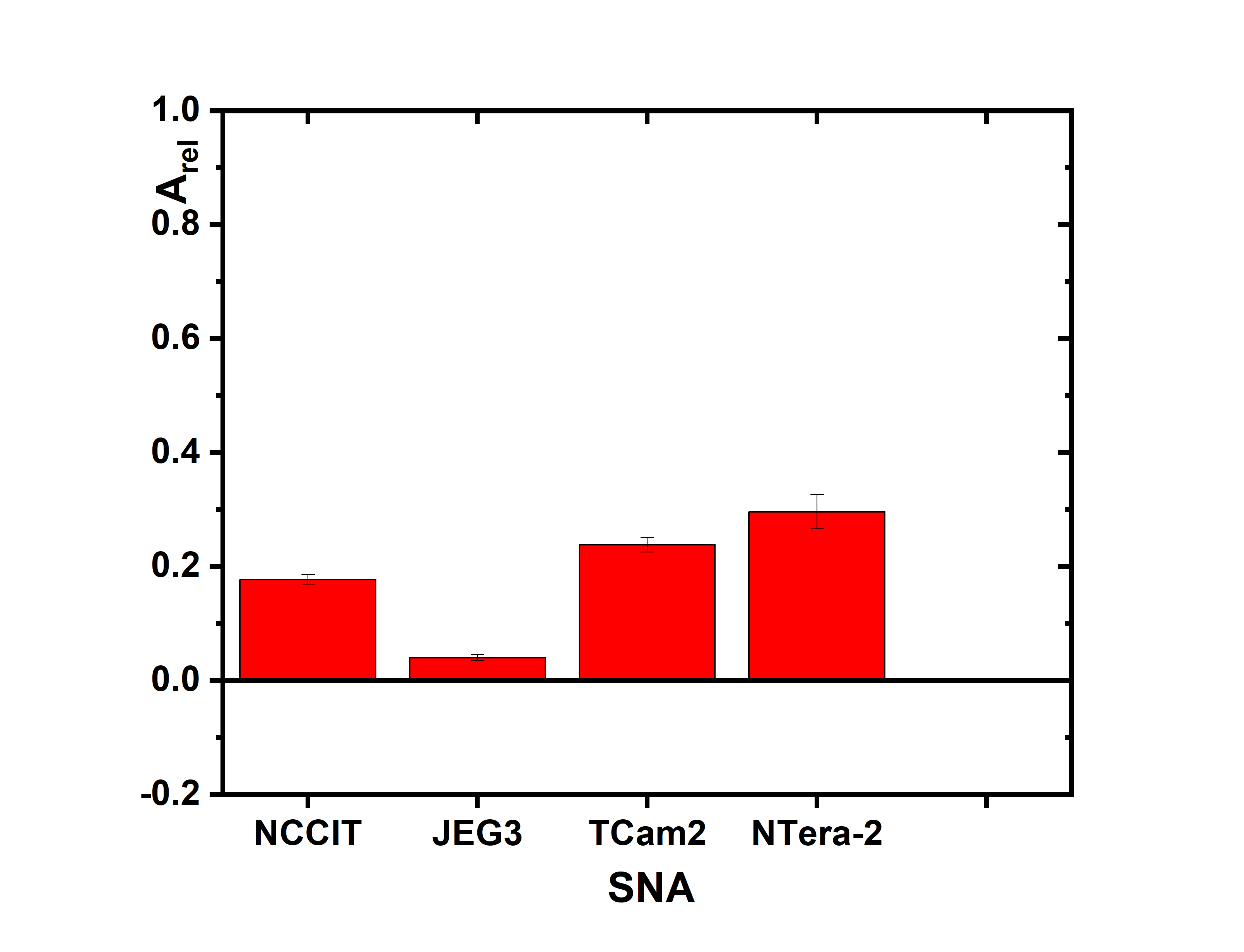

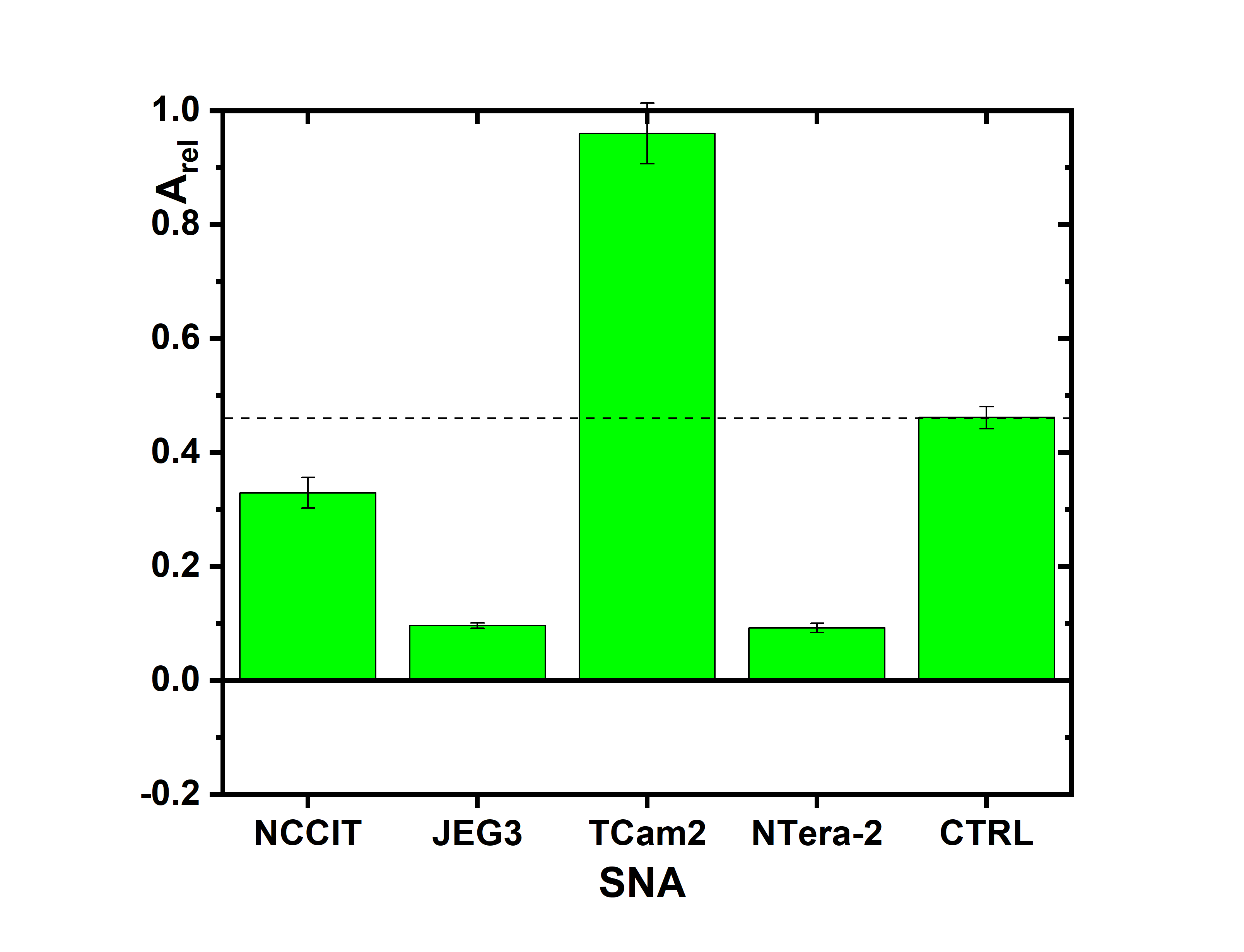
**


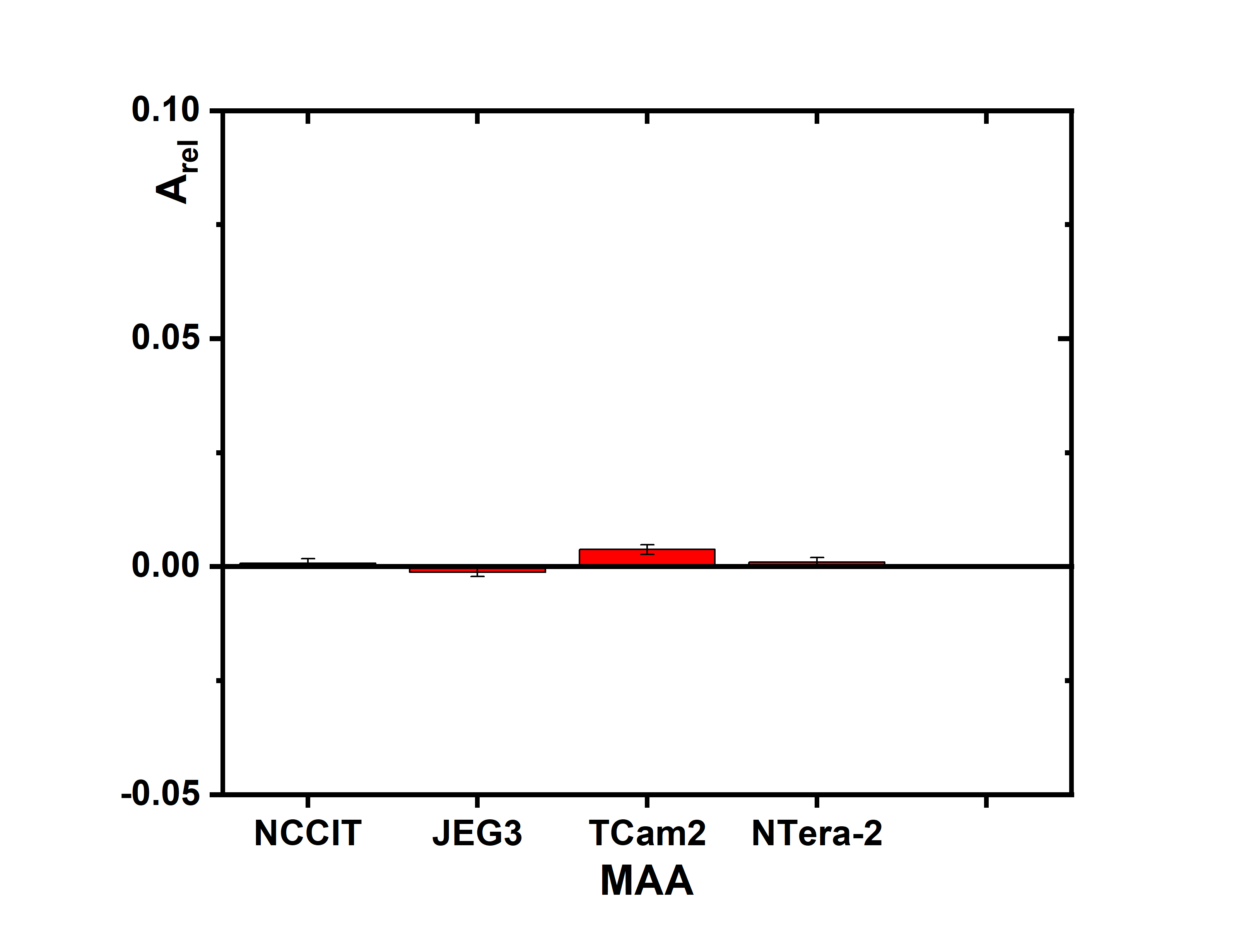

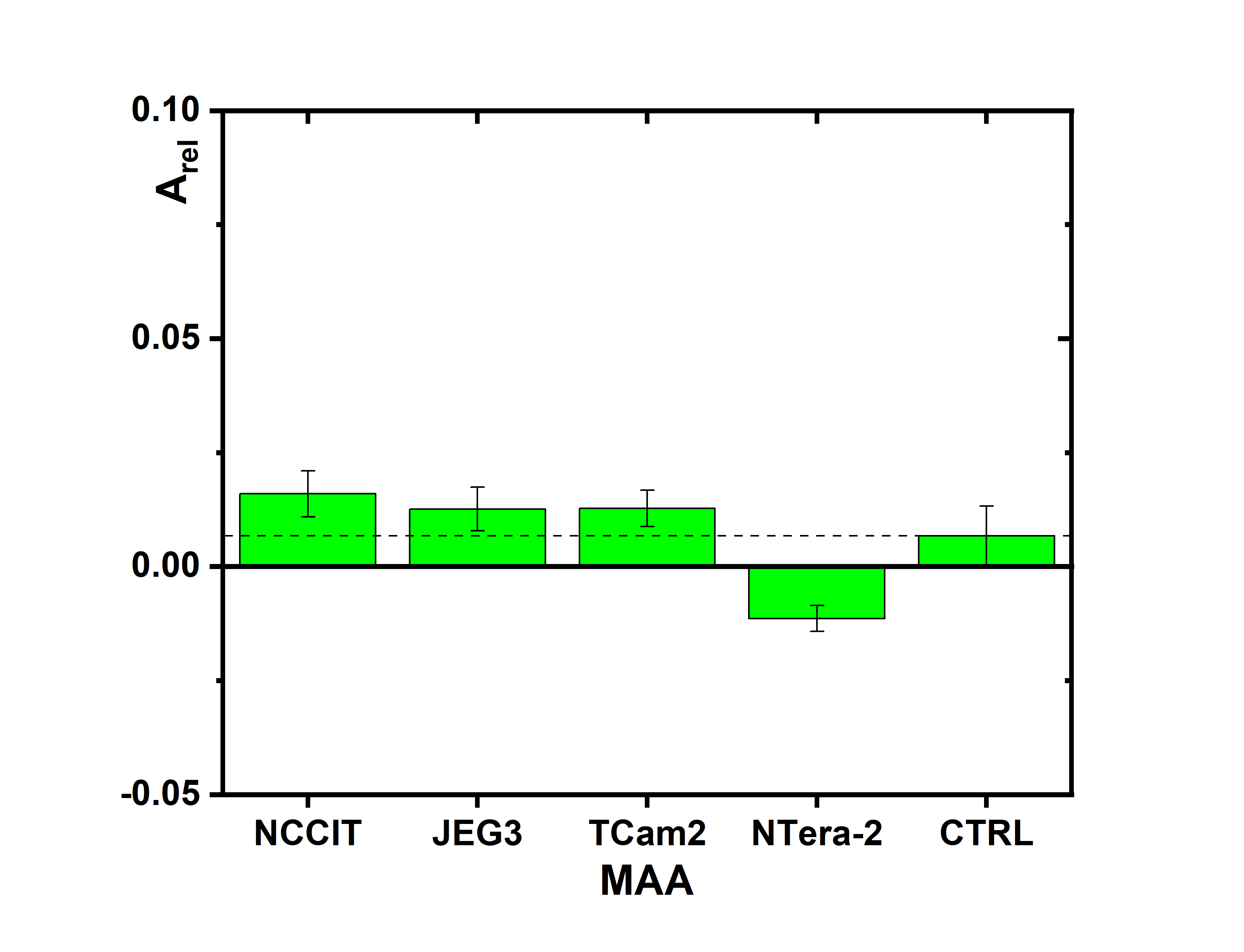


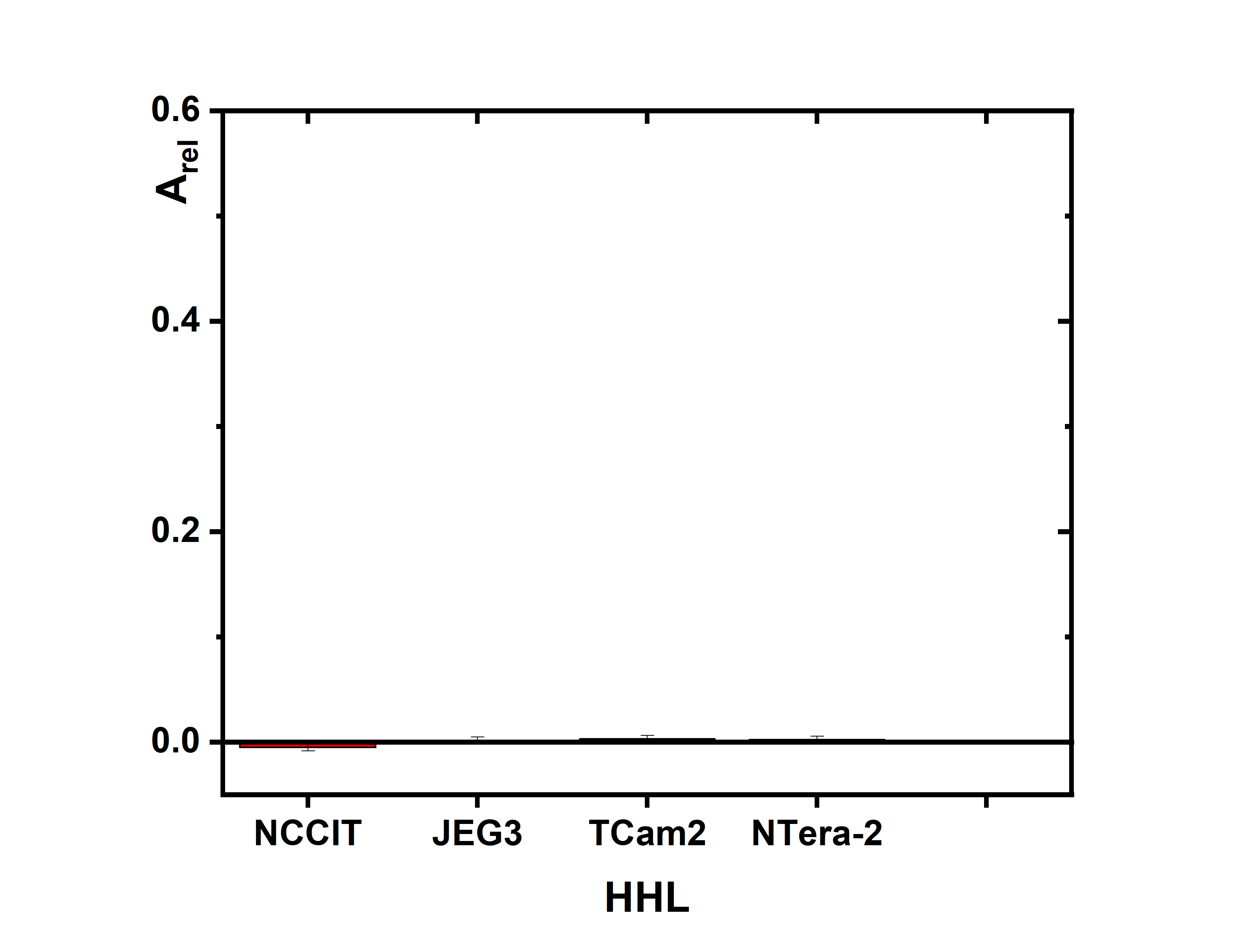

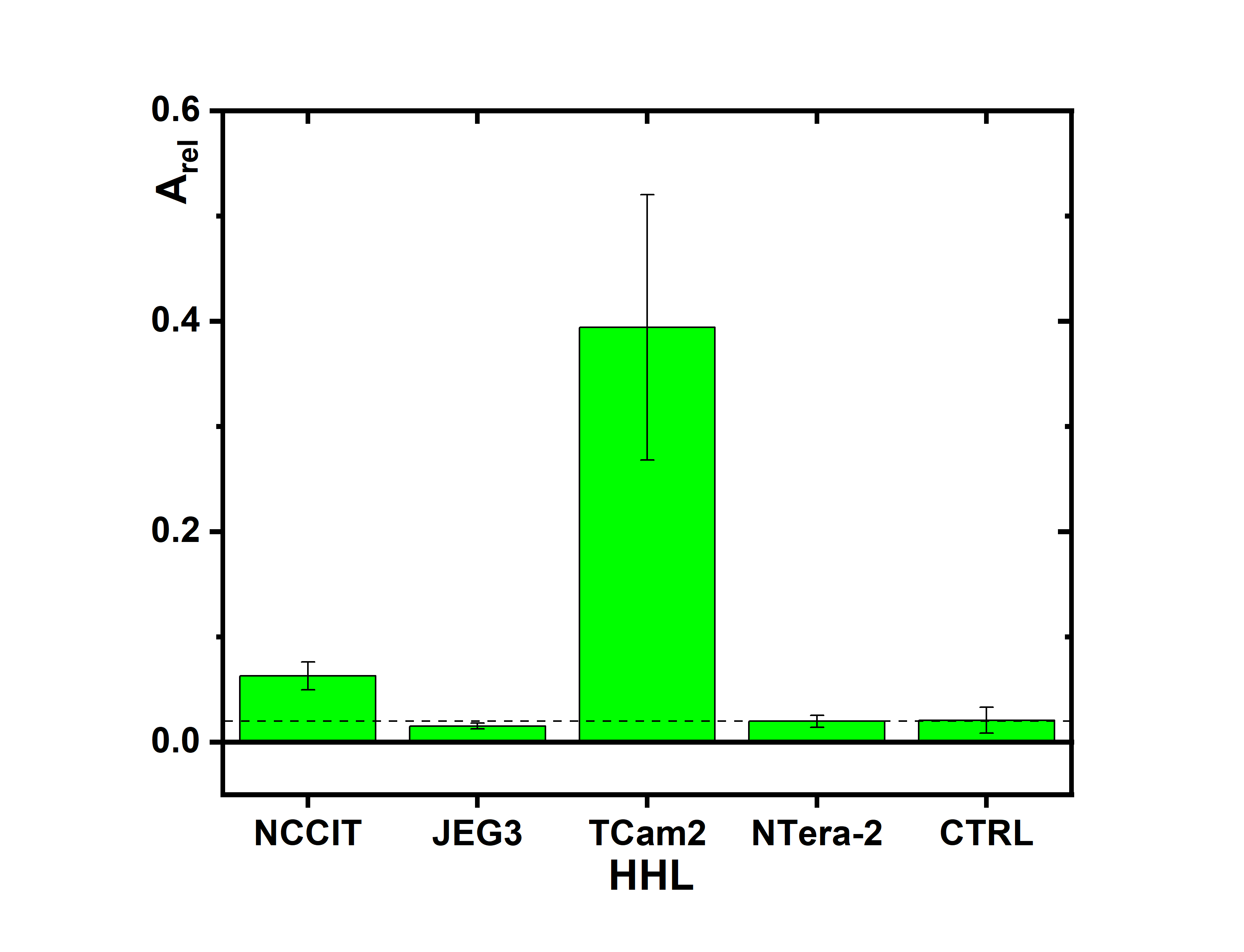


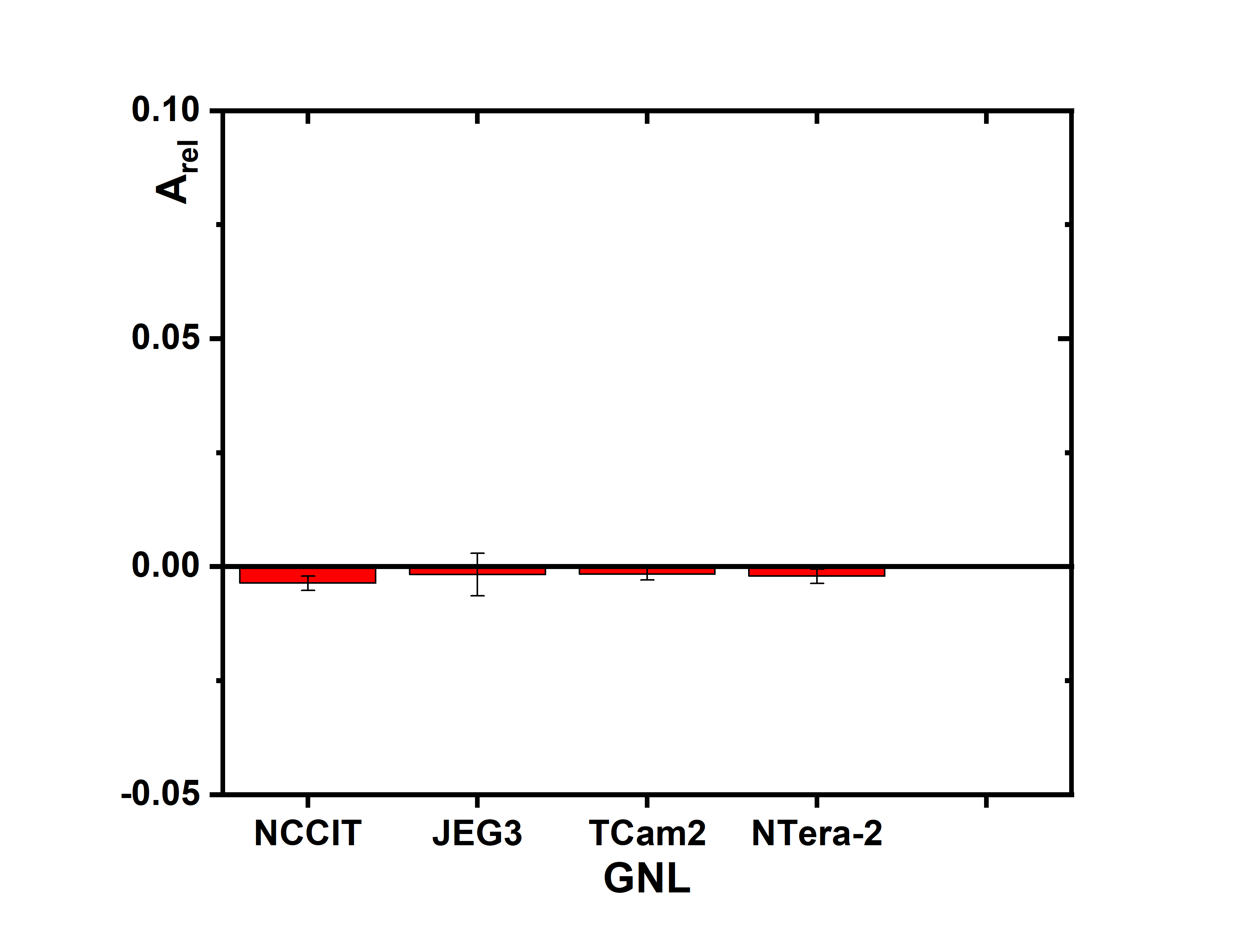

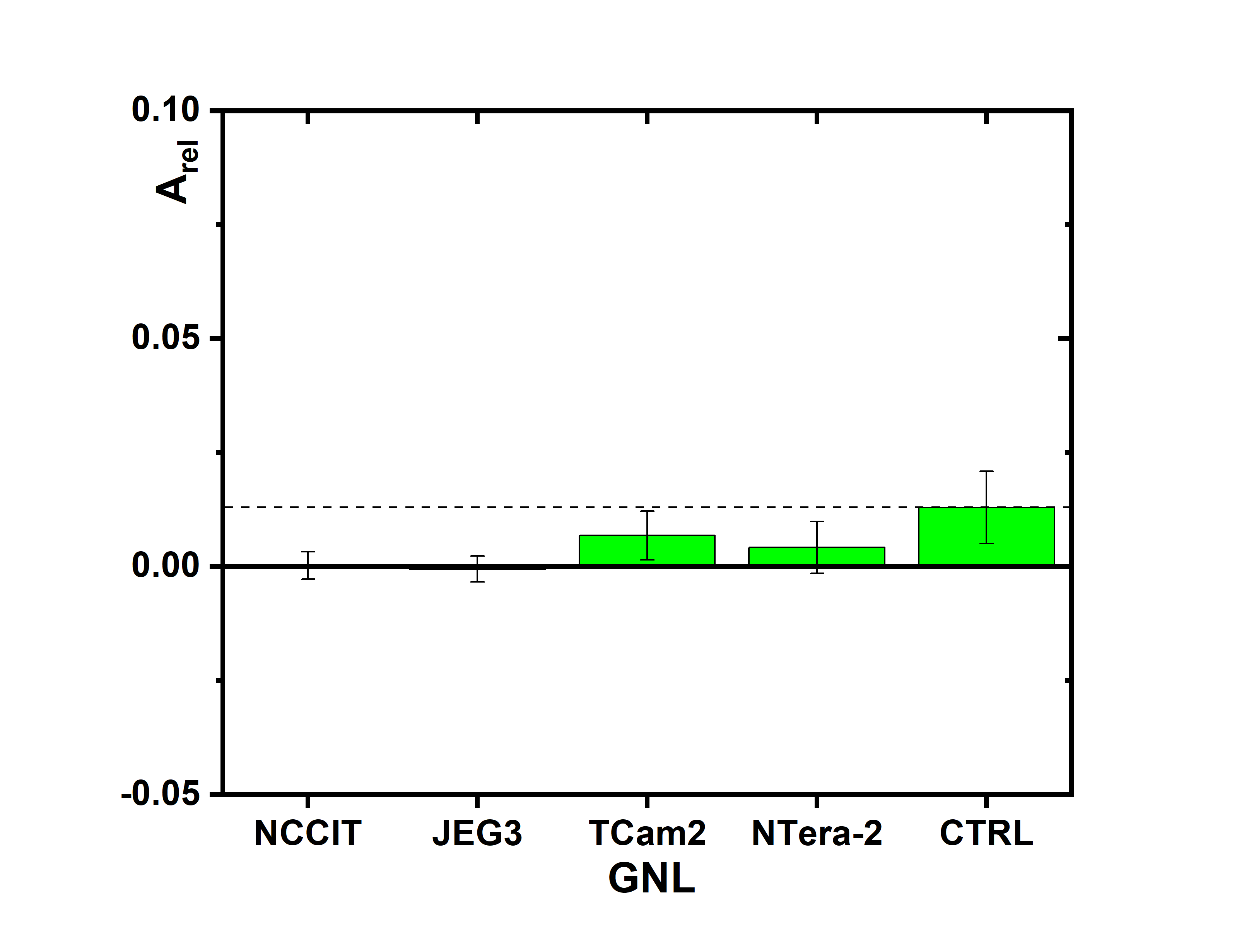


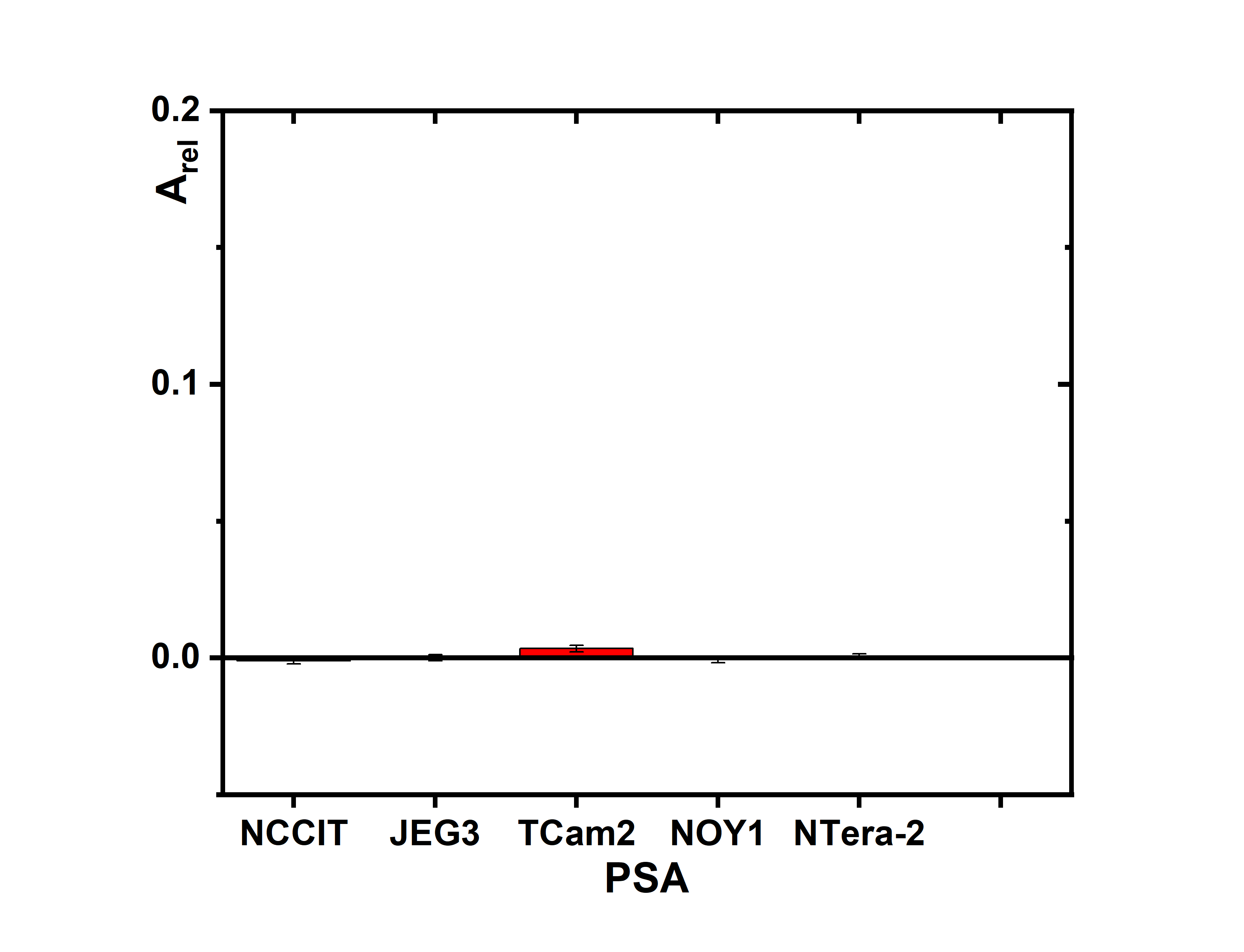

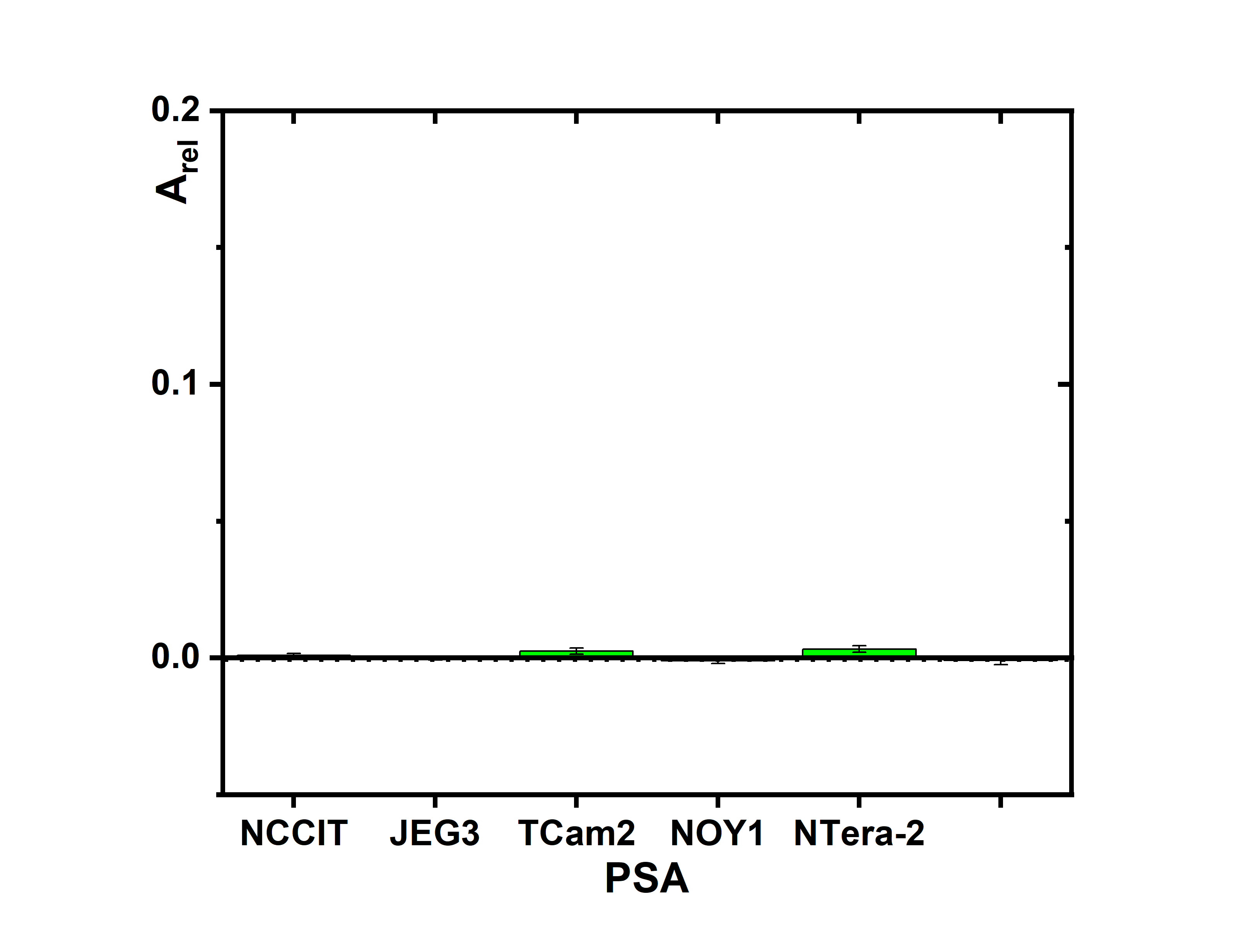


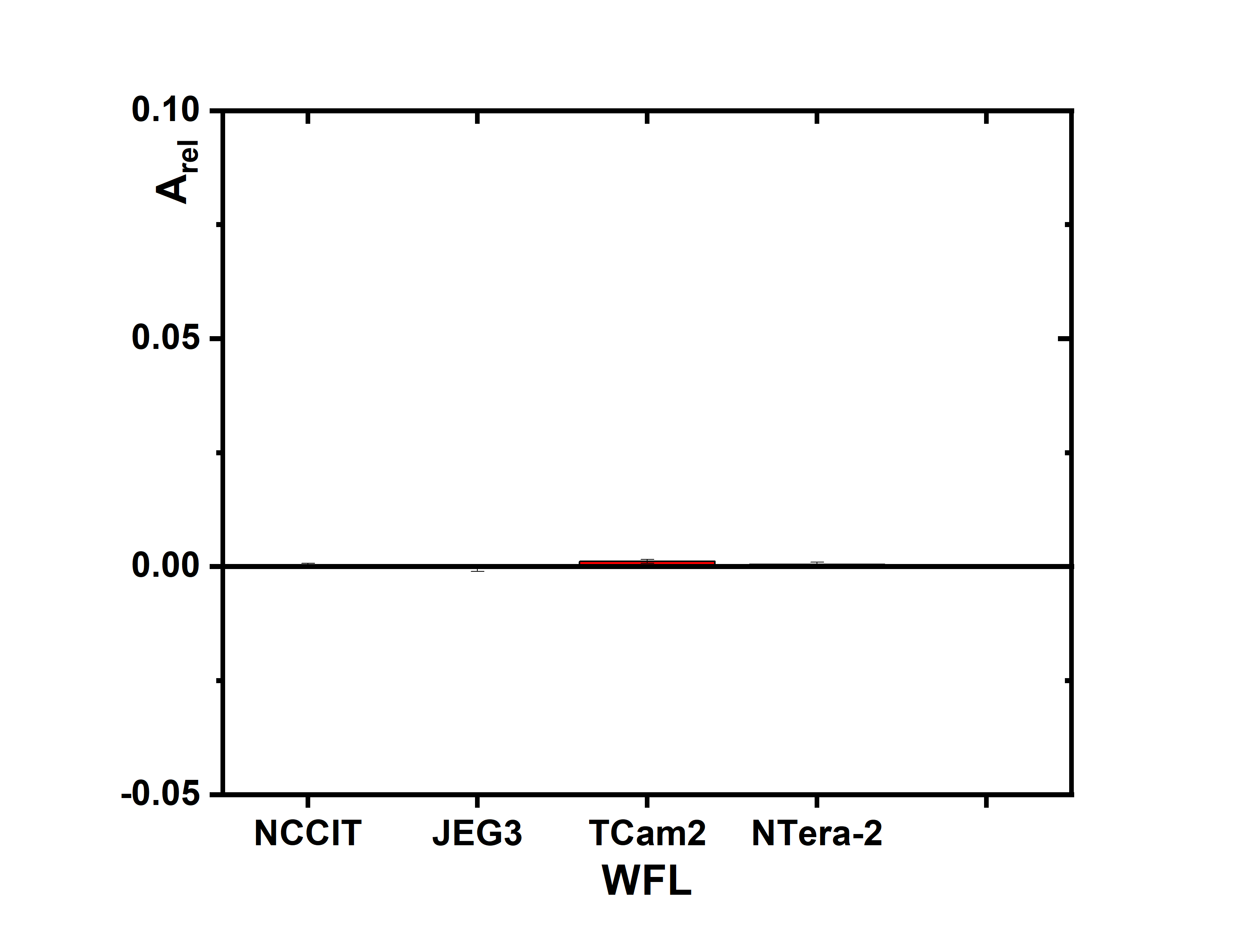

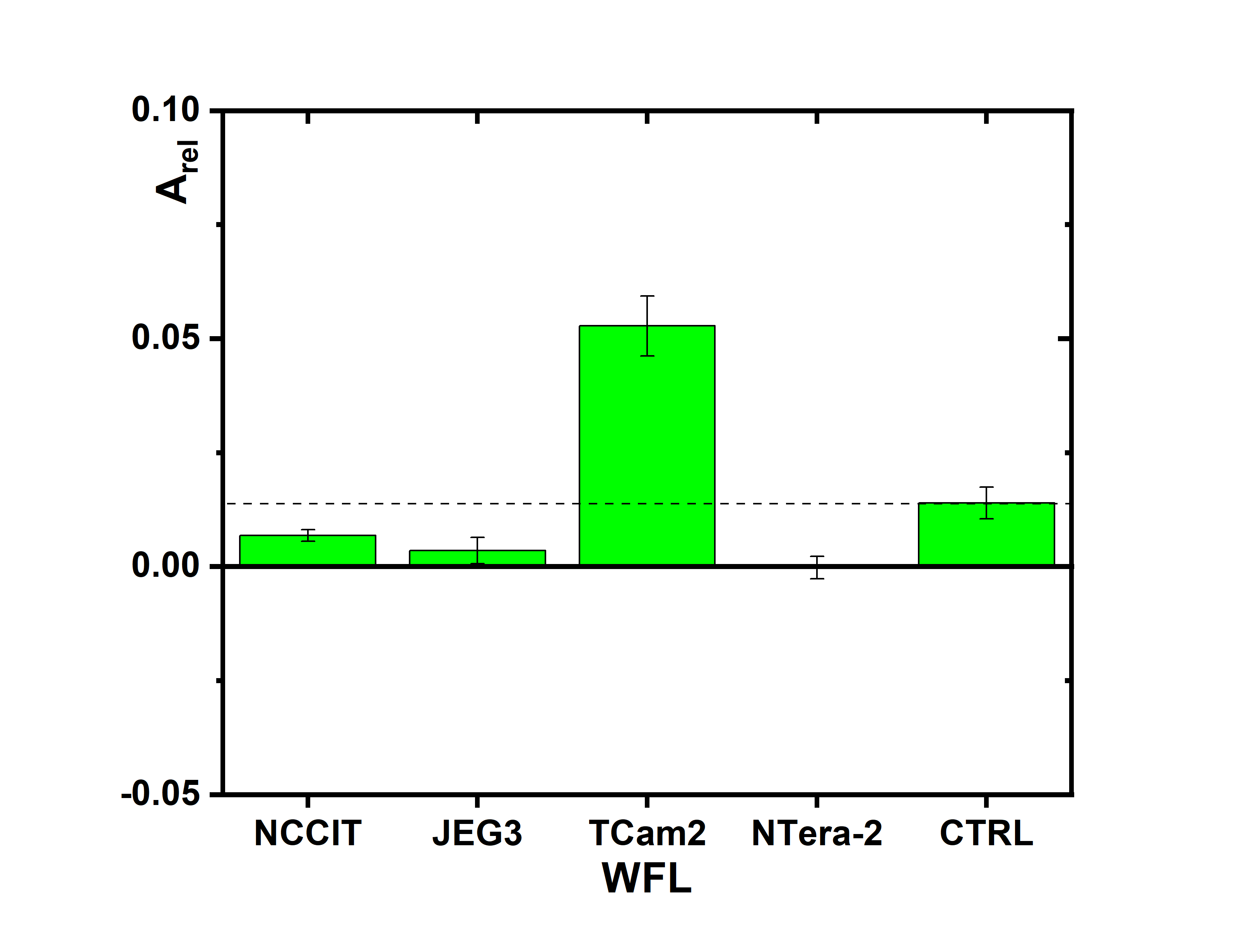


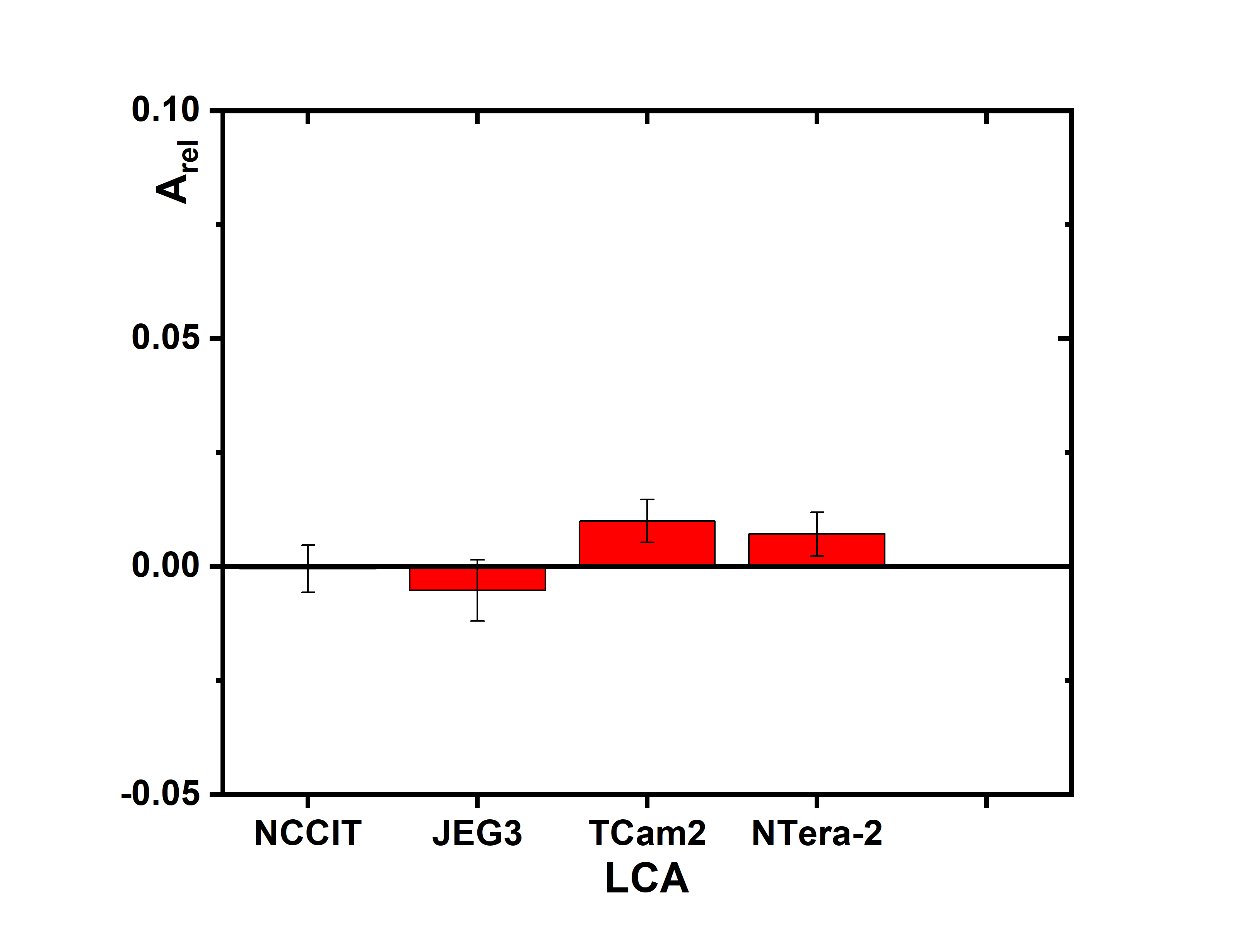

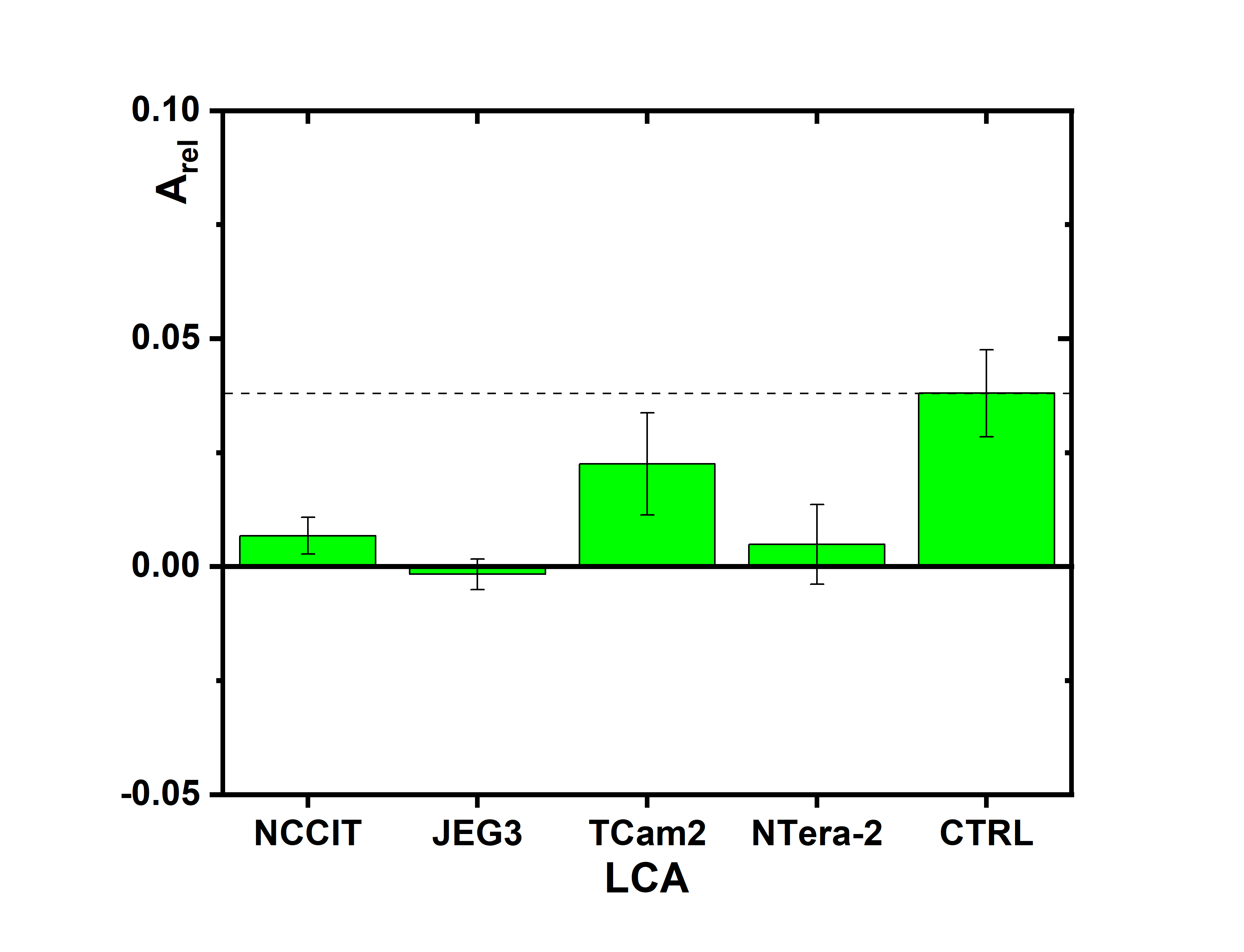


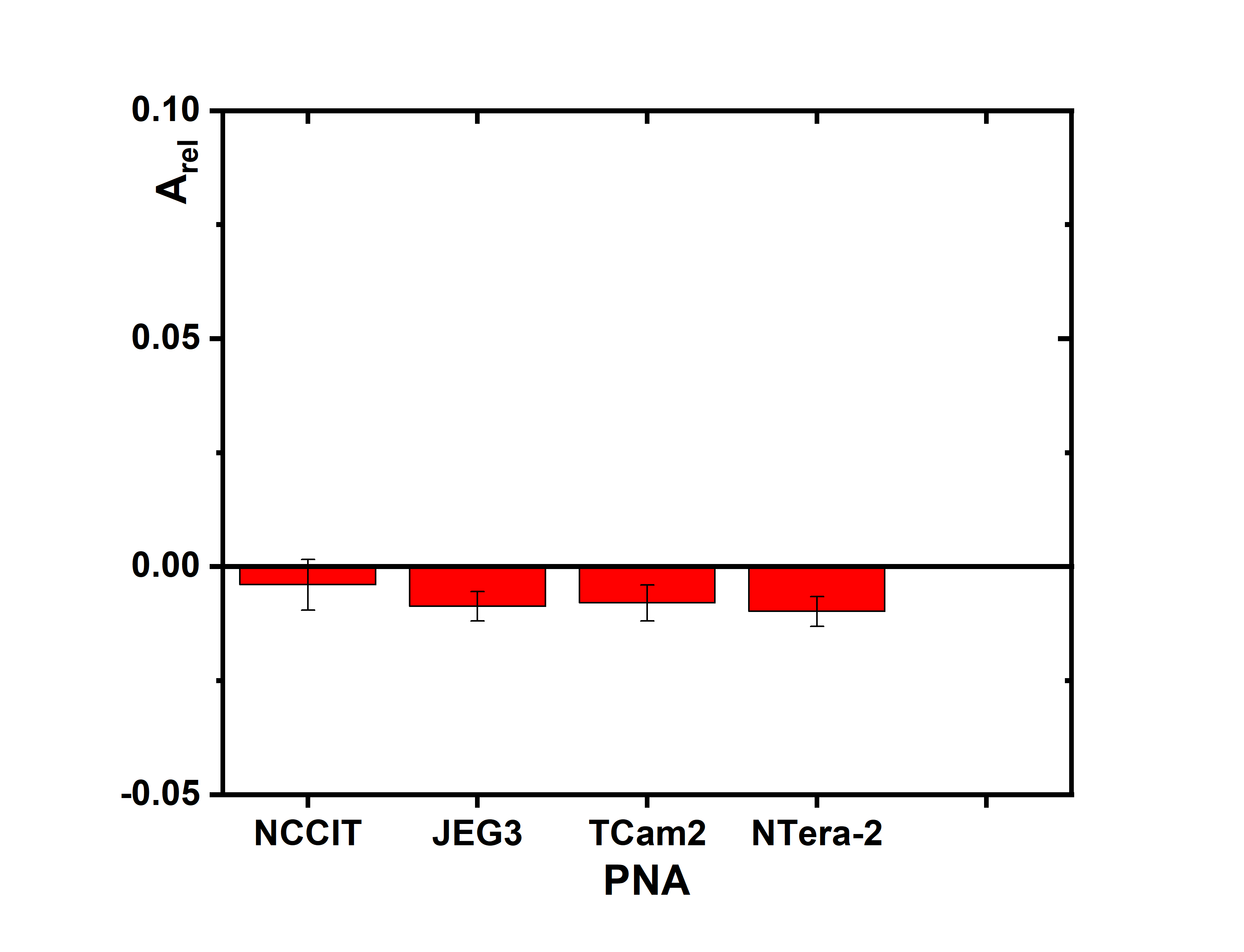

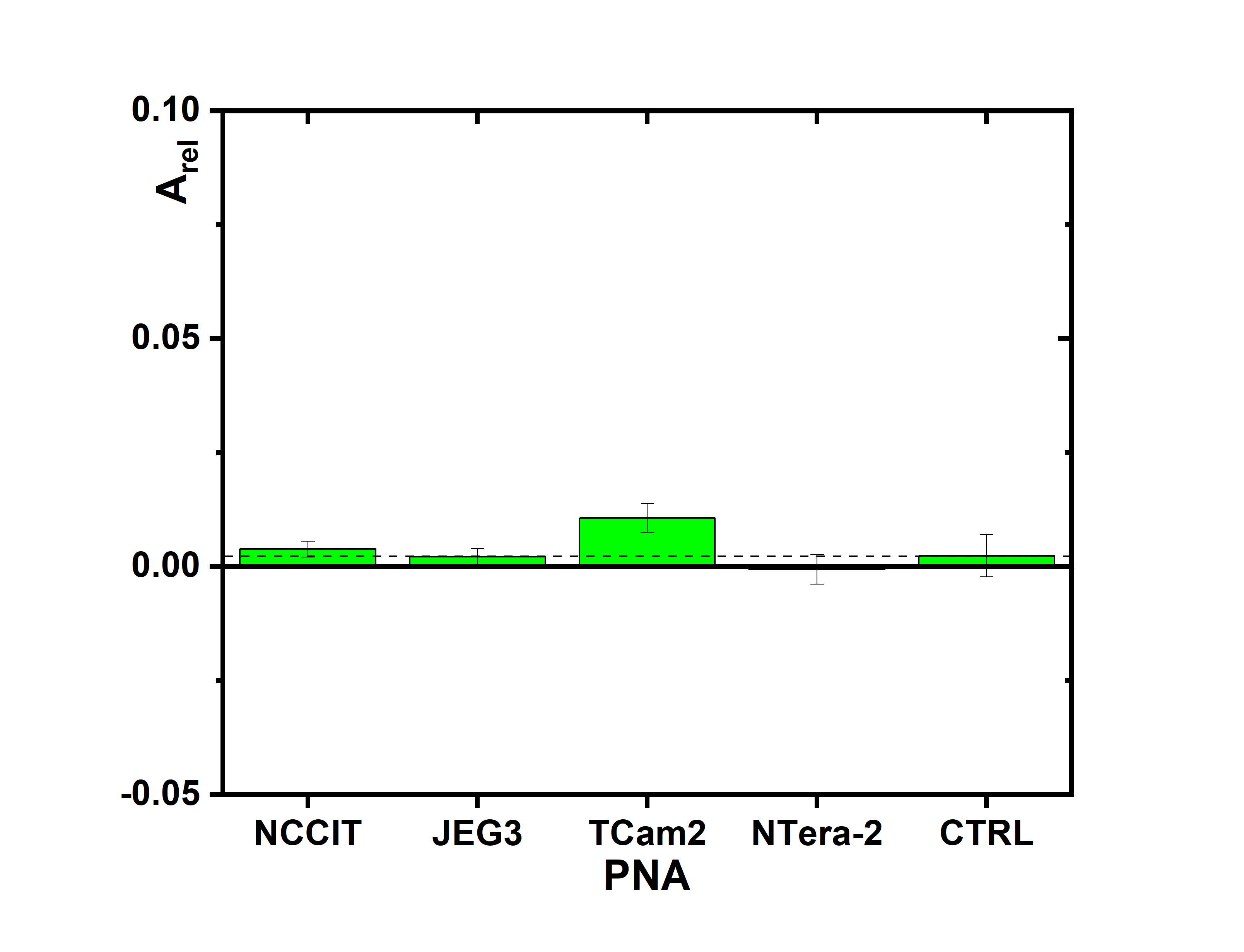


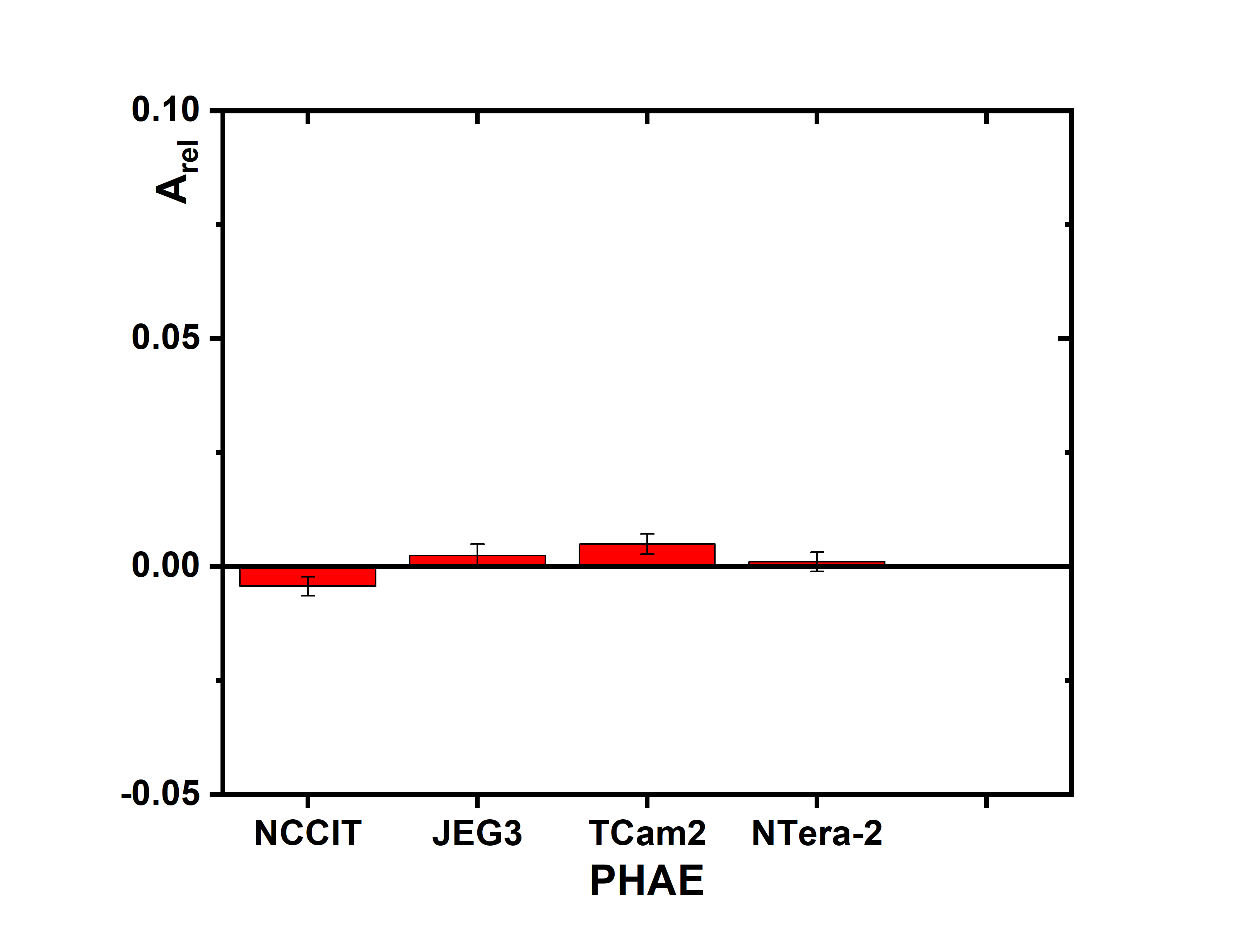

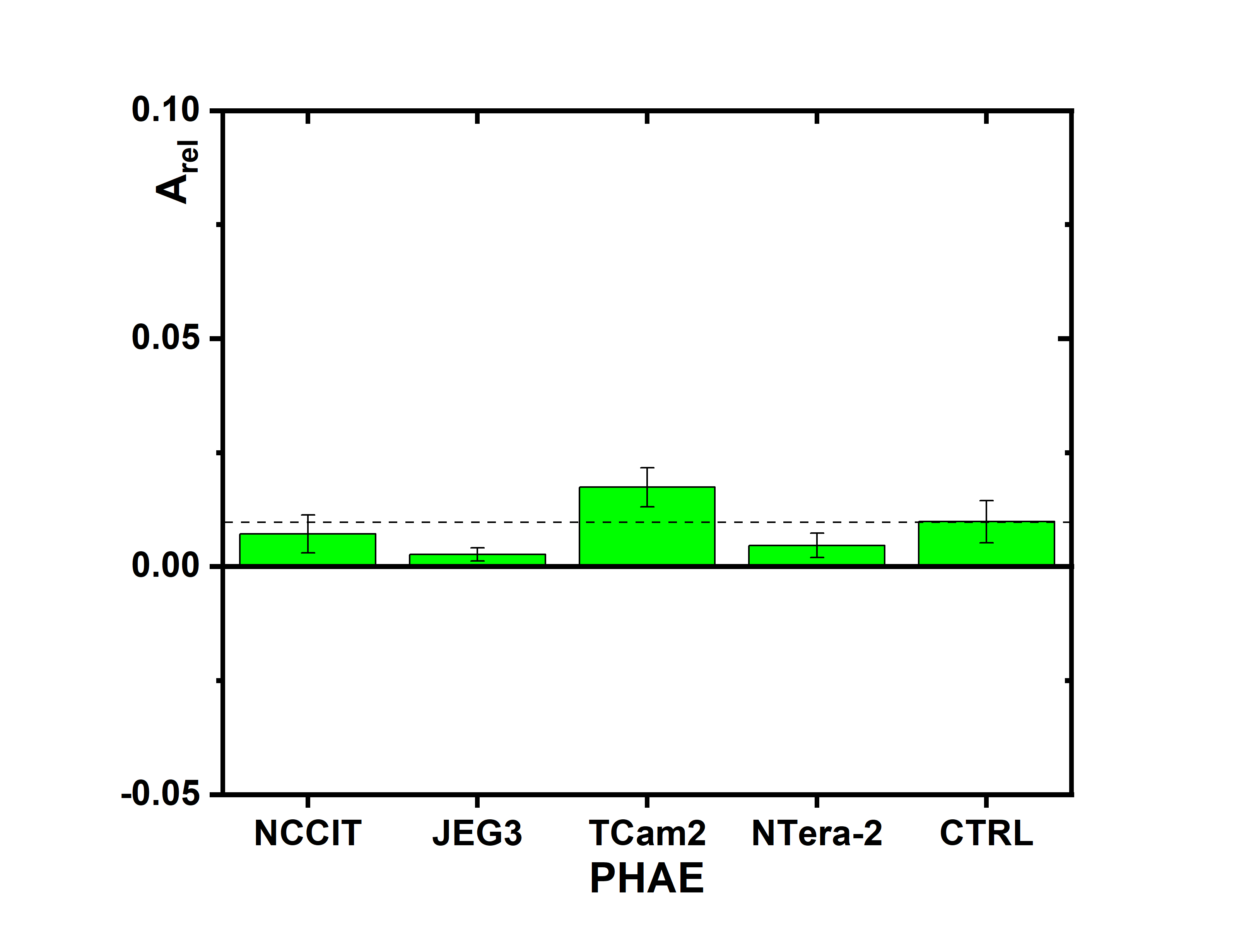


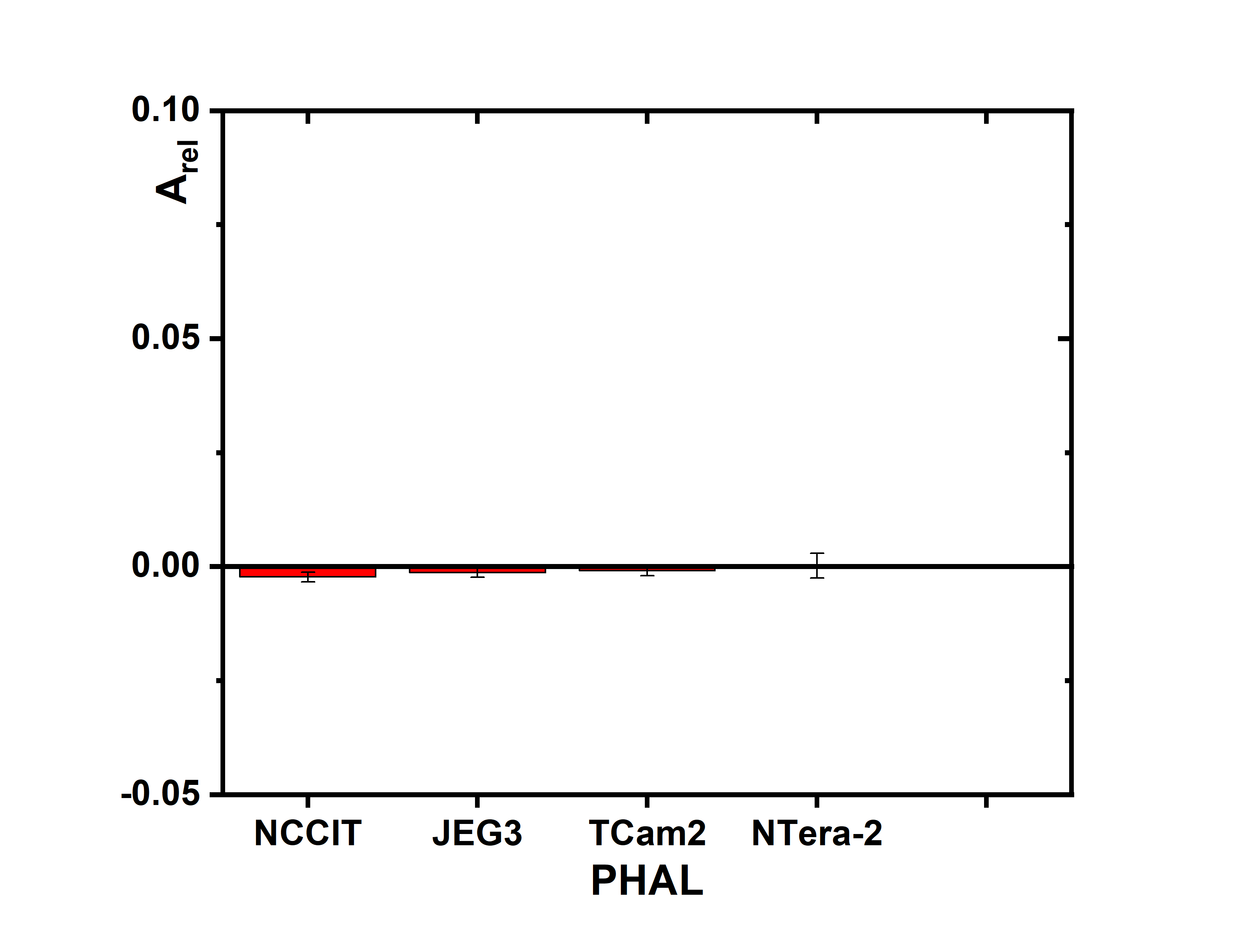

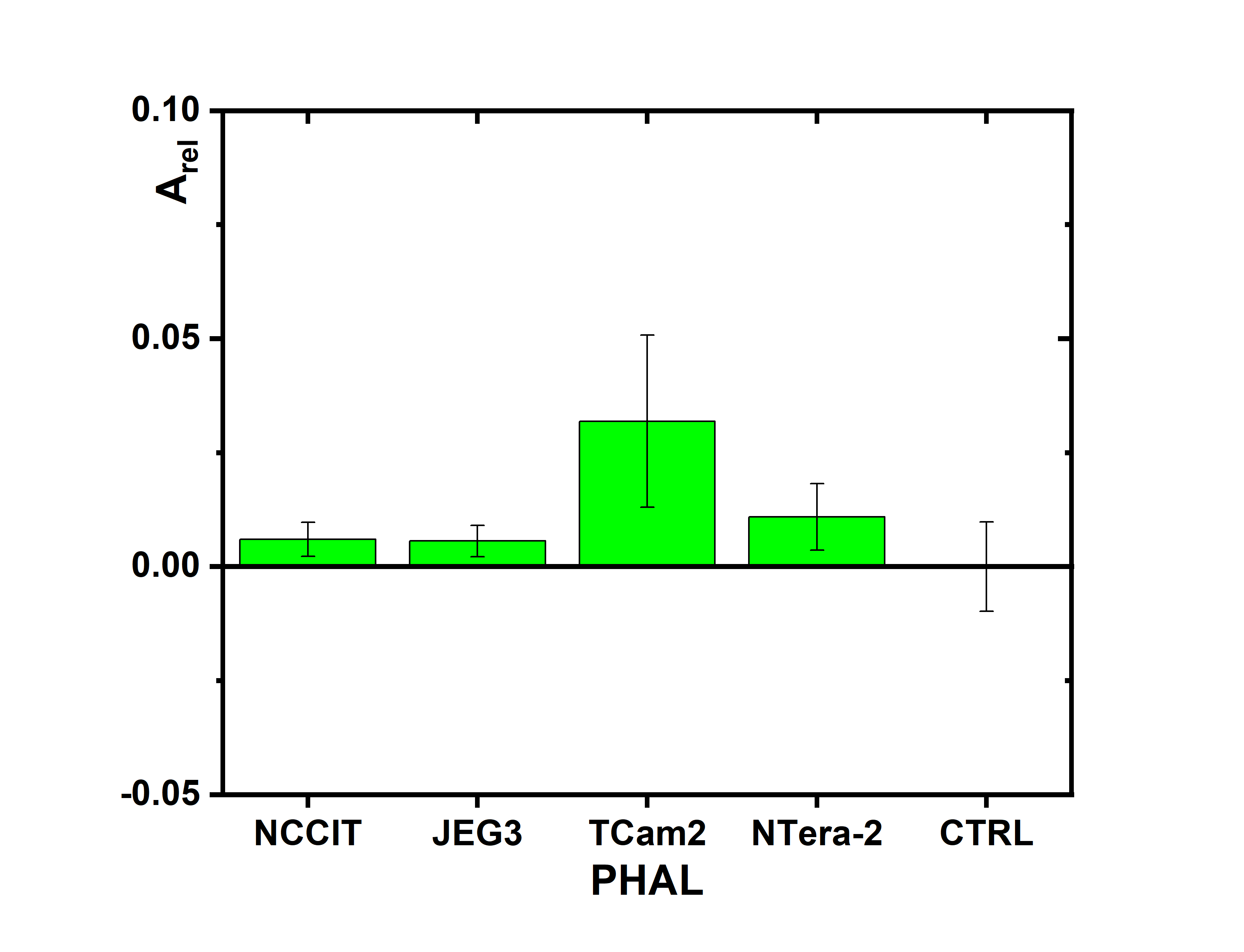


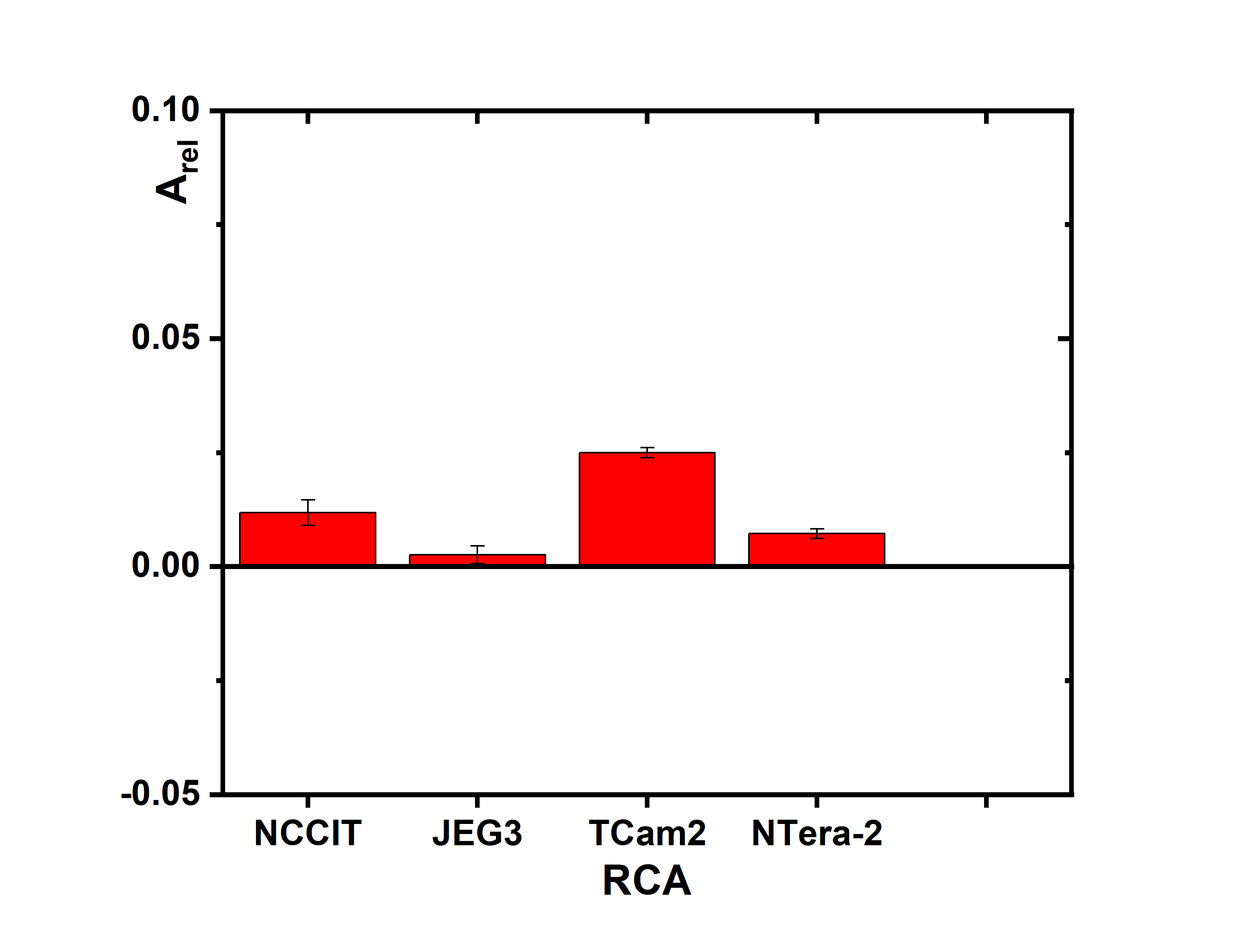

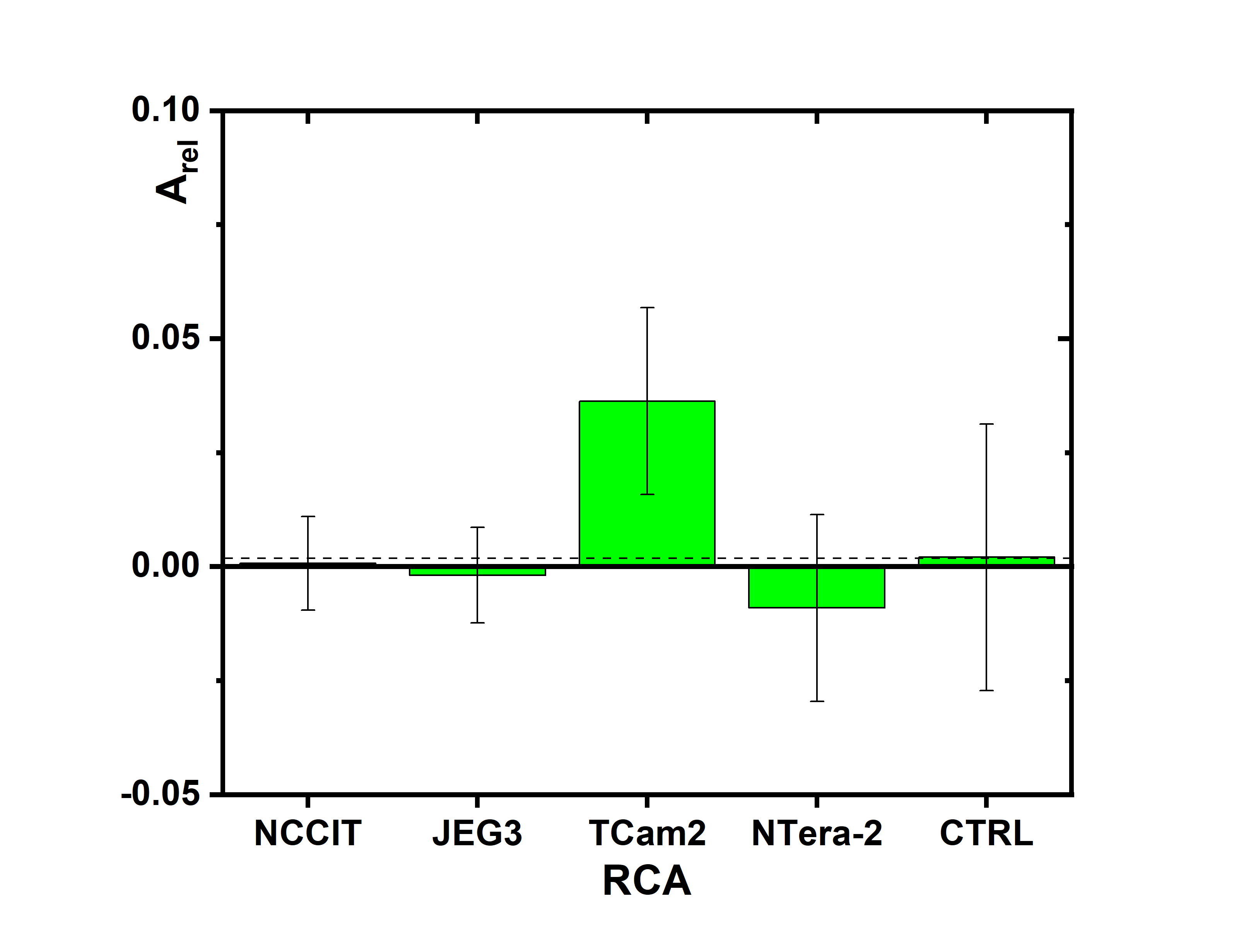


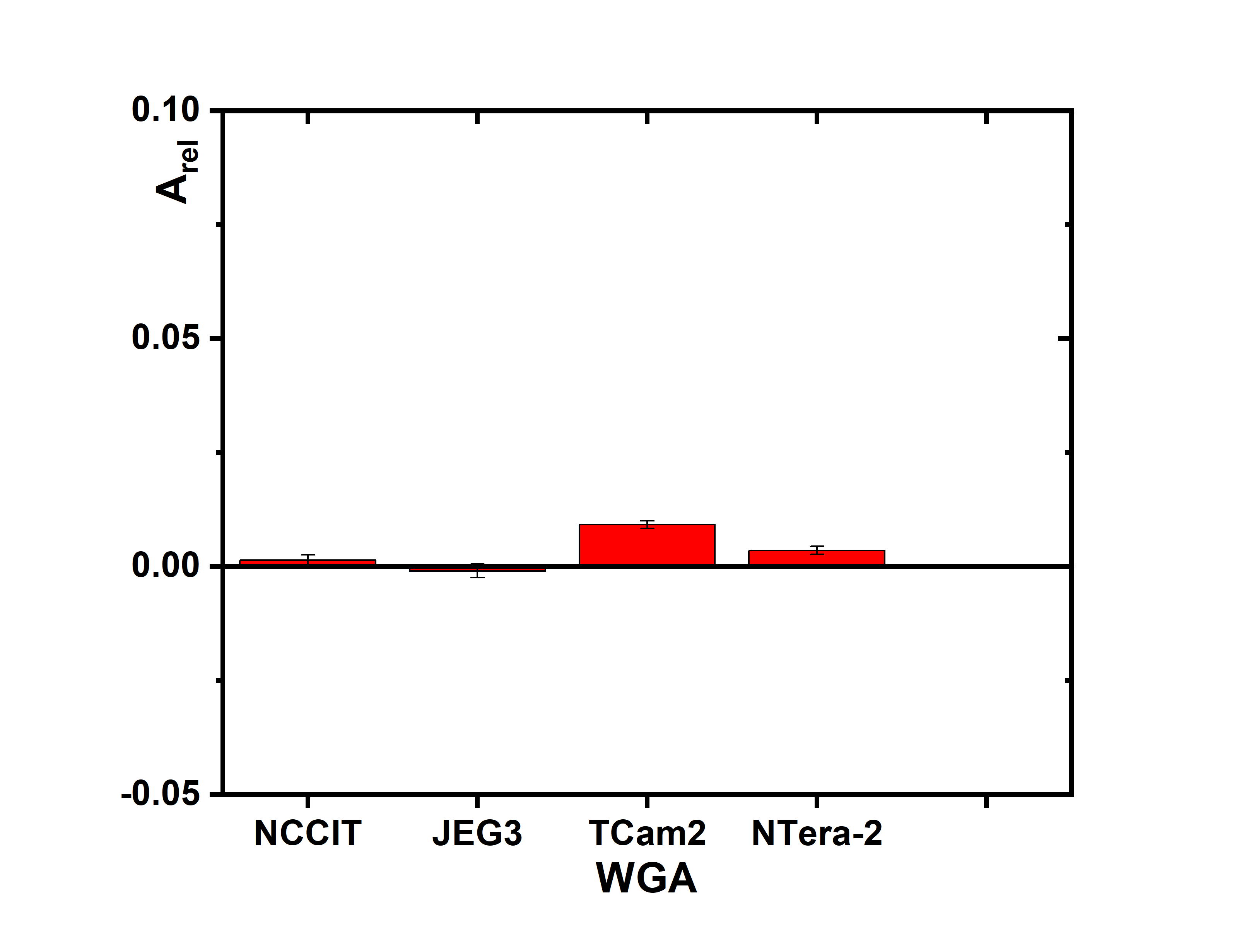

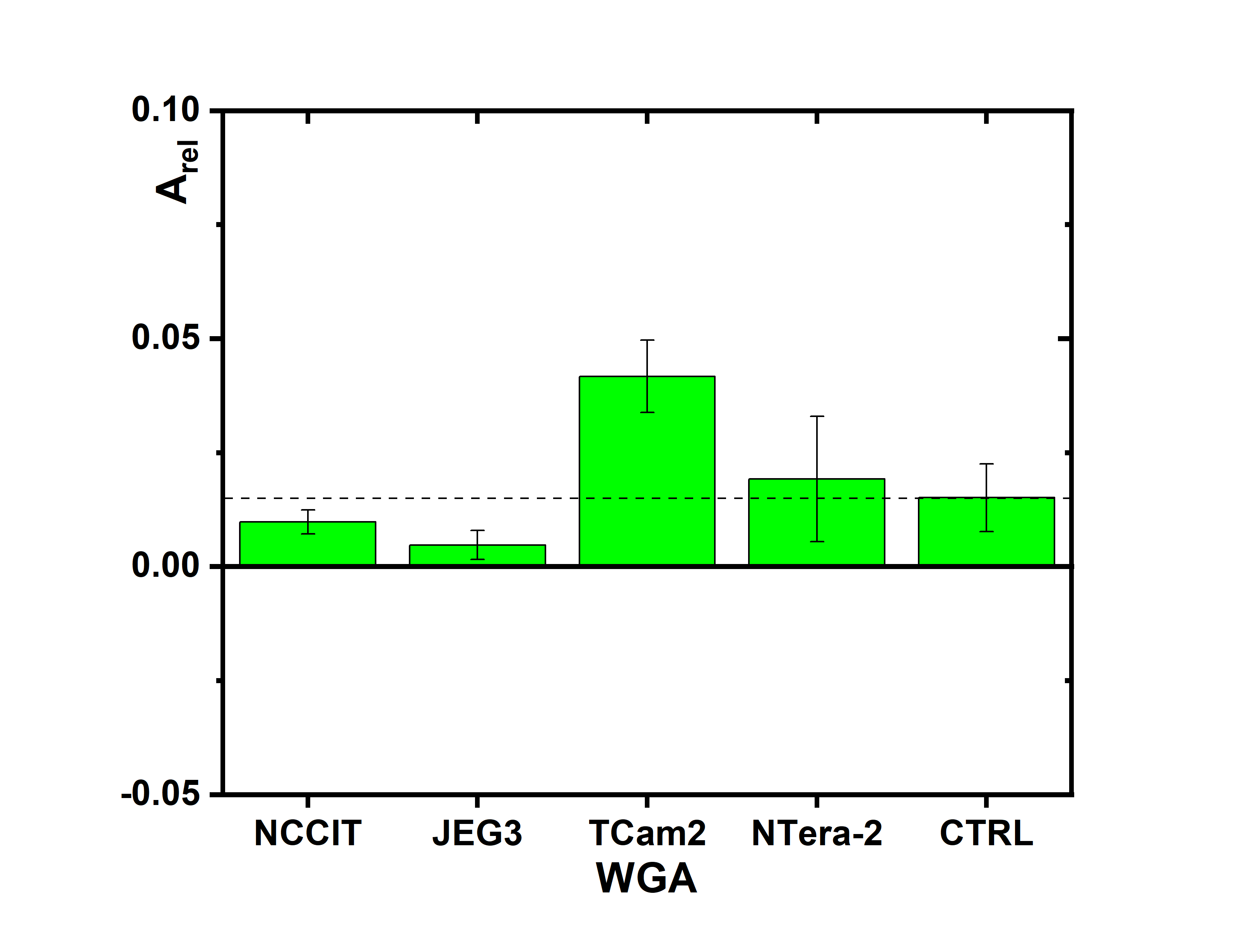


**Figure S2:** Graphs showing binding of 13 lectins to hCG present in the cytosolic fractions of 6 cell lines (sensitive cell lines shown by green bars) or 5 cell lines (cisplatin resistant cell lines shown by red bars) in an ELISA format of analysis. **CTRL** = control cell line CRL/Hs 1.Tes.

**References**

1. Bertok T, Jane E, Chrenekova N, Hroncekova S, Bertokova A, Hires M, Vikartovska A, Kubanikova P, Sokol R, Fillo J. Analysis of serum glycome by lectin microarrays for prostate cancer patients-a search for aberrant glycoforms. *Glycoconjugate J* 2020;**37**: 703-11.

2. Hirabayashi J, Yamada M, Kuno A, Tateno H. Lectin microarrays: concept, principle and applications. *Chem Soc Rev* 2013;**42**: 4443-58.

3. Cummings RD, Darvill AG, Etzler ME, Hahn MG. Glycan-recognizing probes as tools. *Essentials of Glycobiology [internet]* 2017.

4. Houser J, Komarek J, Kostlanova N, Cioci G, Varrot A, Kerr SC, Lahmann M, Balloy V, Fahy JV, Chignard M. A soluble fucose-specific lectin from Aspergillus fumigatus conidia-structure, specificity and possible role in fungal pathogenicity. *PLoS One* 2013;**8**: e83077.

5. Ruhl S, Sandberg A, Cole M, Cisar J. Recognition of immunoglobulin A1 by oral actinomyces and streptococcal lectins. *Infect Immun* 1996;**64**: 5421-4.

6. Läubli H, Borsig L. Selectins promote tumor metastasis. *Seminars Cancer Biol* 2010;**20**: 169-77.

7. Beauharnois ME, Lindquist KC, Marathe D, Vanderslice P, Xia J, Matta KL, Neelamegham S. Affinity and kinetics of sialyl Lewis-X and core-2 based oligosaccharides binding to L-and P-selectin. *Biochemistry* 2005;**44**: 9507-19.

8. Khan ZM, Liu Y, Neu U, Gilbert M, Ehlers B, Feizi T, Stehle T. Crystallographic and glycan microarray analysis of human polyomavirus 9 VP1 identifies N-glycolyl neuraminic acid as a receptor candidate. *J Virol* 2014;**88**: 6100-11.

9. Kobayashi Y, Tateno H, Ogawa H, Yamamoto K, Hirabayashi J. Comprehensive list of lectins: origins, natures, and carbohydrate specificities. *Lectins* 2014: 555-77.

10. Schmidtova S, Kalavska K, Kucerova L. Molecular Mechanisms of Cisplatin Chemoresistance and Its Circumventing in Testicular Germ Cell Tumors. *Curr Oncol Rep* 2018;**20**: DOI: 10.1007/s11912-018-0730-x.
